# New horizons for the therapeutic application of nanozymes in cancer treatment

**DOI:** 10.1186/s12951-025-03185-5

**Published:** 2025-02-20

**Authors:** Pravanjan Malla, Yu-Ming Wang, Chia-Hao Su

**Affiliations:** 1 Center for General Education, Chang Gung University, Taoyuan, 333 Taiwan; 2 Department of Radiation Oncology, Kaohsiung Chang Gung Memorial Hospital, Kaohsiung, 833 Taiwan; 3 Department of Biomedical Imaging and Radiological Sciences, National Yang Ming Chiao Tung University, Taipei, 112 Taiwan

**Keywords:** Nanomaterials, Reactive-oxygen species, Therapy

## Abstract

The advent of nanozymes has revolutionized approaches to cancer diagnosis and therapy, introducing innovative strategies that address the limitations of conventional treatments. Nanozyme nanostructures with enzyme-mimicking catalytic abilities exhibit exceptional stability, biocompatibility, and customizable functions, positioning them as promising tools for cancer theranostics. By emulating natural enzyme reactions, nanozymes can selectively target and eradicate cancer cells, minimizing harm to adjacent healthy tissues. Nanozymes can also be functionalized with specific targeting ligands, allowing for the precise delivery and regulated release of therapeutic agents, improving treatment effectiveness and reducing adverse effects. However, issues such as biocompatibility, selectivity, and regulatory compliance remain critical challenges for the clinical application of nanozymes. This review provides an overview of nanozymes, highlighting their unique properties, various classifications, catalytic activities, and diverse applications in cancer treatments. The strategic oncological deployment of nanozymes could profoundly impact future advancements in personalized medicine, highlighting recent progress and prospective directions in enzyme-mimetic approaches for cancer treatment. This review summarizes an overview of nanozymes, highlighting their unique properties, various classifications, catalytic activities, and diverse applications in cancer treatments.

**Graphical Abstract:**

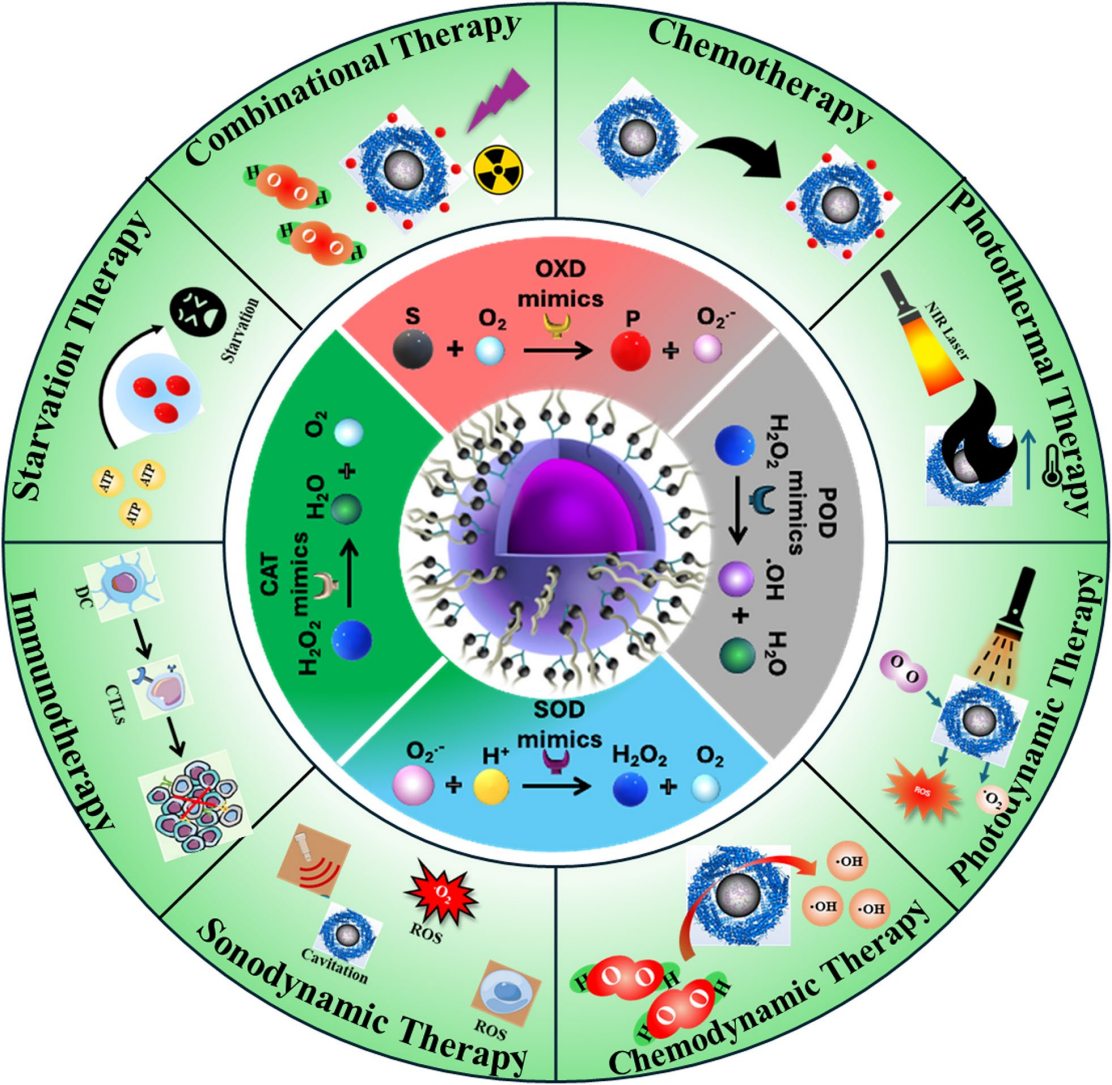

## Highlights

- A brief history of the discovery and development of nanozymes.
- Physicochemical properties, classification, and catalytic mechanism.
- Overview of cancer diagnosis and the potential synergistic effects of cancer therapies.

## Introduction to nanozymes

**Fig. 1:**
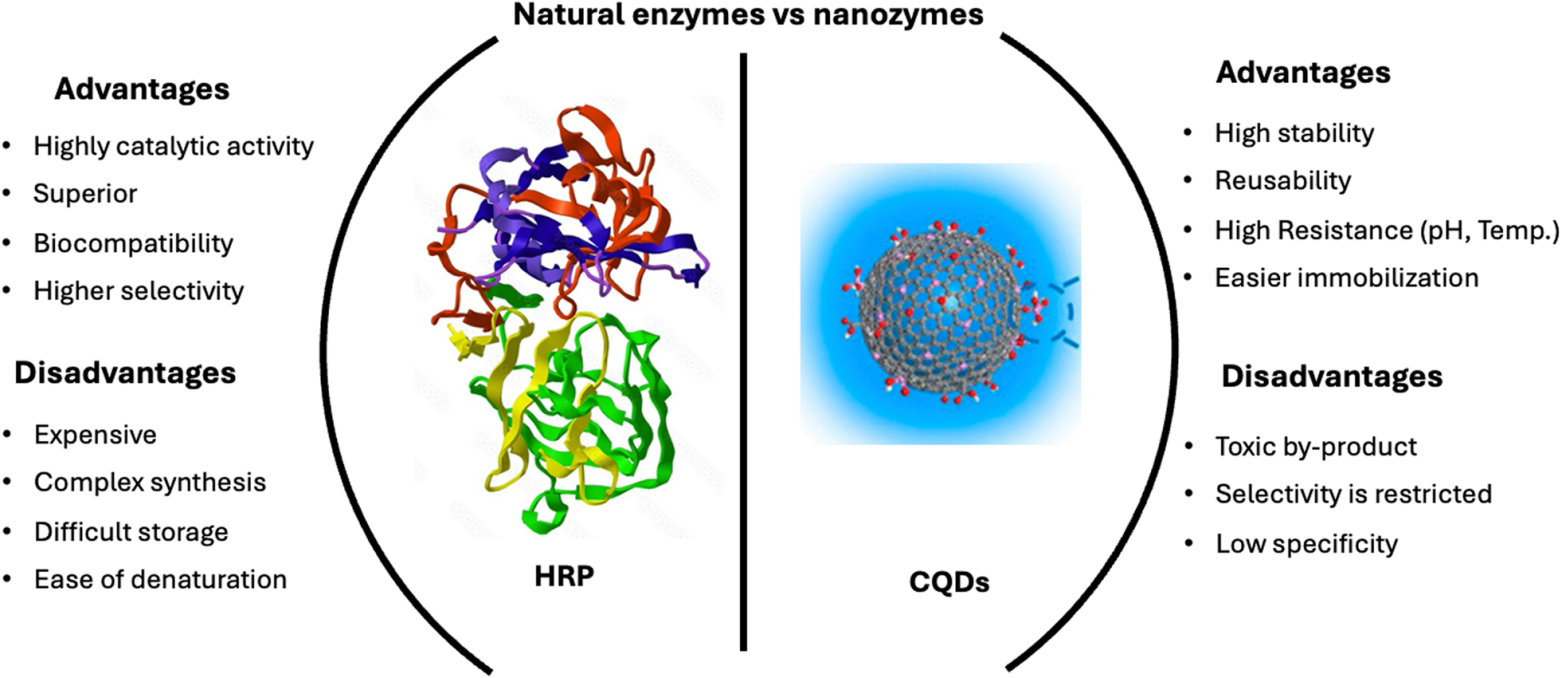
Comparison of natural enzymes and nanozymes. Whereas HRP-Horseradish peroxidase, CQDs-Carbon quantum dots

Nanozymes are nanomaterials that possess intrinsic enzyme-like properties, functioning as artificial enzymes [1, 2]. Their unique characteristics, such as their small size, site-specific targeting, and atomic structure, make them highly promising [3, 4]. These properties enable site-specific targeting, significantly advancing nanobiotechnology in cancer treatment. Research on nanozymes began in the 1990s, and the term “nanozyme” was introduced by Scrimin et al. in 2004 [5]. The field gained significant attention in 2007 [6], when Yan et al. discovered that ferromagnetic nanoparticles possess enzyme-like activity. Since then, considerable research attention has focused on nanozymes, producing a wide variety of these nanomaterials that possess intrinsic enzyme-like catalytic activity, enabling them to effectively mimic the functions of natural enzymes while overcoming their limitations including instability, high cost, and intricate production processes [7, 8]. These materials typically range in size from 1 to 100 diameter and can be composed of diverse materials, including metals, metal oxides, and carbon-based compounds [9]. They boast unique properties including high stability, ease of modification, adjustable catalytic activity, functional diversity, recyclability, feasibility for large-scale preparation, and cost-effectiveness, making them attractive alternatives to traditional enzymes and therapeutic agents [10–12]. Furthermore, some nanozymes possess unique physicochemical properties, such as magnetism, photothermal qualities, and photodynamic effects, which can be remotely controlled via a magnetic field, laser, ultrasound, or heat [13]. The comparison between natural enzymes and nanozymes is illustrated in Fig. 1. In therapeutic applications, nanozymes hold significant promise for disease treatment and management. They can be designed to target specific biological pathways, facilitating the delivery of therapeutic agents directly to diseased tissues while minimizing side effects [14, 15]. For example, researchers have examined the potential application of nanozymes in cancer therapy, where they can increase the efficacy of chemotherapeutic drugs or serve as agents for photothermal therapy by generating heat upon light exposure [16].

Moreover, nanozymes can play crucial roles in combating oxidative stress, a common factor in various diseases, including neurodegenerative disorders and cardiovascular diseases. By mimicking antioxidant enzymes, nanozymes can help neutralize harmful reactive oxygen species (ROS), thereby protecting cells from damage and promoting overall health [17, 18]. As research continues to advance, the therapeutic applications of nanozymes are expected to expand, offering innovative solutions for a range of medical conditions and paving the way for more effective and targeted treatment strategies. The versatility and adaptability of nanozymes position them as a promising frontier in the development of next-generation therapeutics.

This comprehensive review delves into the fascinating world of nanozymes, highlighting their distinctive characteristics that set them apart from traditional enzymes. It explores the different classifications of nanozymes, discussing their various structural and functional features. Additionally, the review examines the catalytic activities exhibited by these innovative biomimetic catalysts and illustrates their diverse applications, particularly in the realm of cancer treatment, showcasing the promising potential they hold in improving therapeutic strategies.

### History and development

**Fig. 2:**
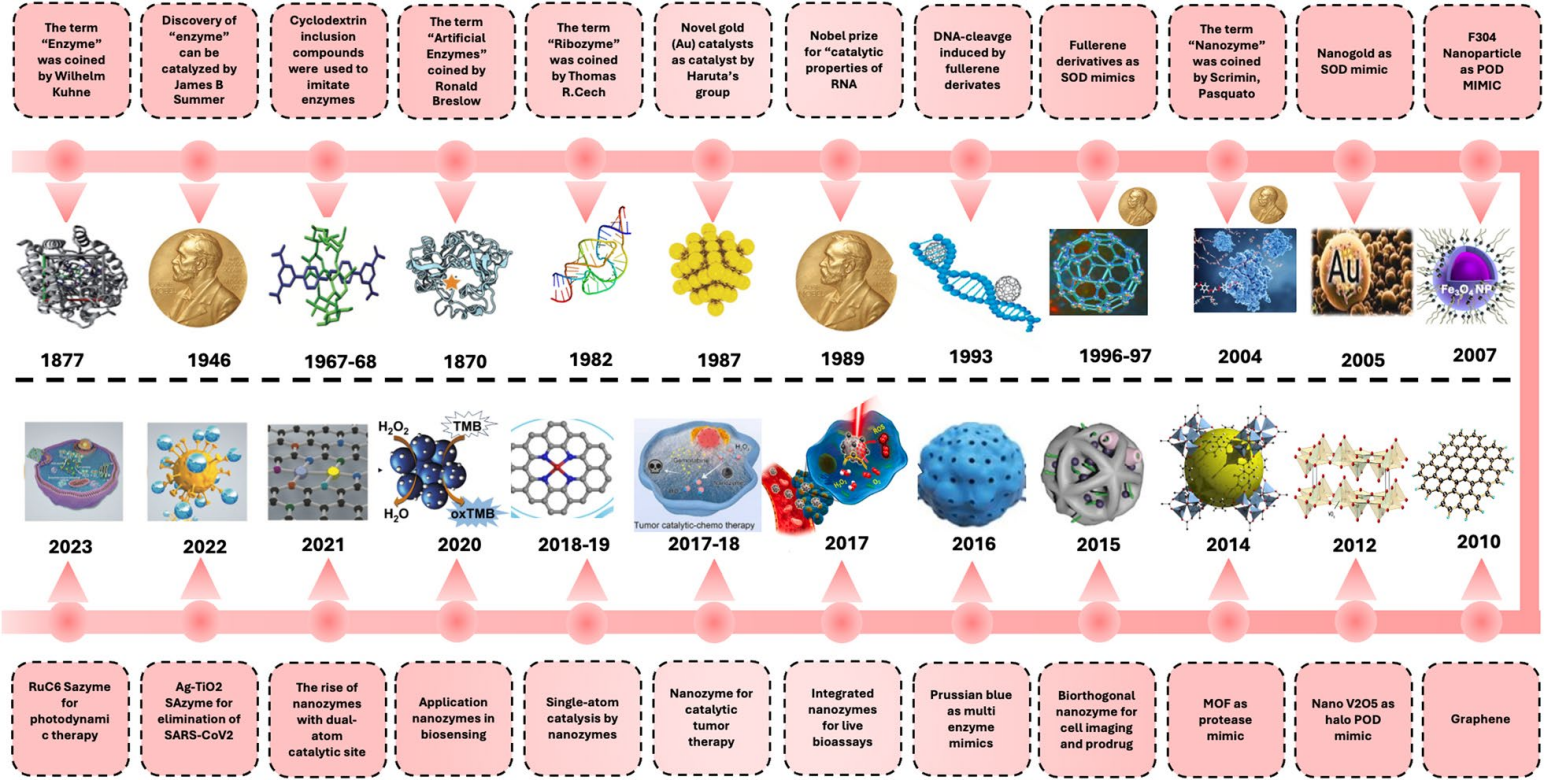
Chronological overview of key milestones in the historical progression of nanozyme research, focusing on atomic site innovations and catalytic therapeutic applications. Reproduced with permission from [32]

The discovery and development of nanozymes represent significant advances at the intersection of nanotechnology and enzymology, raising innovative solutions to challenges in various fields, including biomedicine, environmental science, and catalysis [19]. The focused exploration of nanozymes began in the early 2000s when researchers first reported that certain nanoparticles, particularly metal and metal oxide nanoparticles, could catalyze reactions similar to those of natural enzymes. The term "nanozyme" was introduced by Pasquato and his team in 2004 following their experimental discovery of the ribonuclease-mimicking activity exhibited by thiol-protected gold nanoparticles [20, 21]. This groundbreaking revelation opened new avenues for designing and applying nanomaterials, leading to a surge of interest in their potential as enzyme substitutes. The unique properties of nanozymes, including high catalytic stability, ease of functionalization, and low production cost, have made them attractive candidates for various applications, from drug delivery systems to biosensors and environmental remediation [22, 23]. These characteristics make them particularly appealing for applications where traditional enzymes may not be viable. The extensive history of the discovery and development of nanozymes is presented in Fig. 2.

More recently, research has focused on enhancing the catalytic efficiency and substrate specificity of nanozymes through various synthesis and modification techniques. This led to the development of diverse nanozyme types, each tailored for specific applications, particularly in the realm of cancer therapy and diagnostics [24]. Despite their promise, the field has faced challenges, including issues related to their low activity levels compared to natural enzymes and the need for improved targeting capabilities. These nanozymes can be engineered to have specific catalytic activities, allowing the creation of custom solutions for various biochemical reactions [25, 26]. For example, metal nanoparticles, metal oxides, and carbon-based nanomaterials have been found to exhibit peroxidase, catalase, and oxidase-like activity. Complex biological environments pose significant hurdles to effective in vivo nanozyme applications, including issues related to biocompatibility, metabolic stability, and potential toxicity [27]. Ongoing research is focused on optimizing nanozyme design to increase their selectivity and minimize adverse effects, paving the way for their safe and effective use in clinical settings. With recent advances in nanotechnology, many nanomaterials have shown unexpected enzyme-like activities [28]. This versatility has enabled researchers to explore a wide range of applications, from environmental sensing to medical diagnostics. Today, the field of nanozymes continues to evolve, driven by ongoing research aimed at addressing these challenges and unlocking their full potential. With advancements in materials science and a deeper understanding of the structure–activity relationships of nanozymes, the future holds exciting possibilities for their integration into cutting-edge technologies and personalized medicine. As we explore the history and development of nanozymes, it is becoming increasingly clear that they are not just a novel scientific curiosity but also a transformative tool with the potential to revolutionize various industries.

Moreover, the integration of nanozymes into biosensing platforms has revolutionized the way we monitor biological processes and detect biomarkers [29–31]. The ability to create highly sensitive and selective sensors using nanozymes has opened new avenues for early disease detection, the real-time monitoring of physiological conditions, and personalized medicine. For example, wearable sensors based on nanozymes can continuously monitor glucose levels in diabetic patients, providing critical data for effective disease management.

### Physicochemical properties of nanozymes

**Fig. 3:**
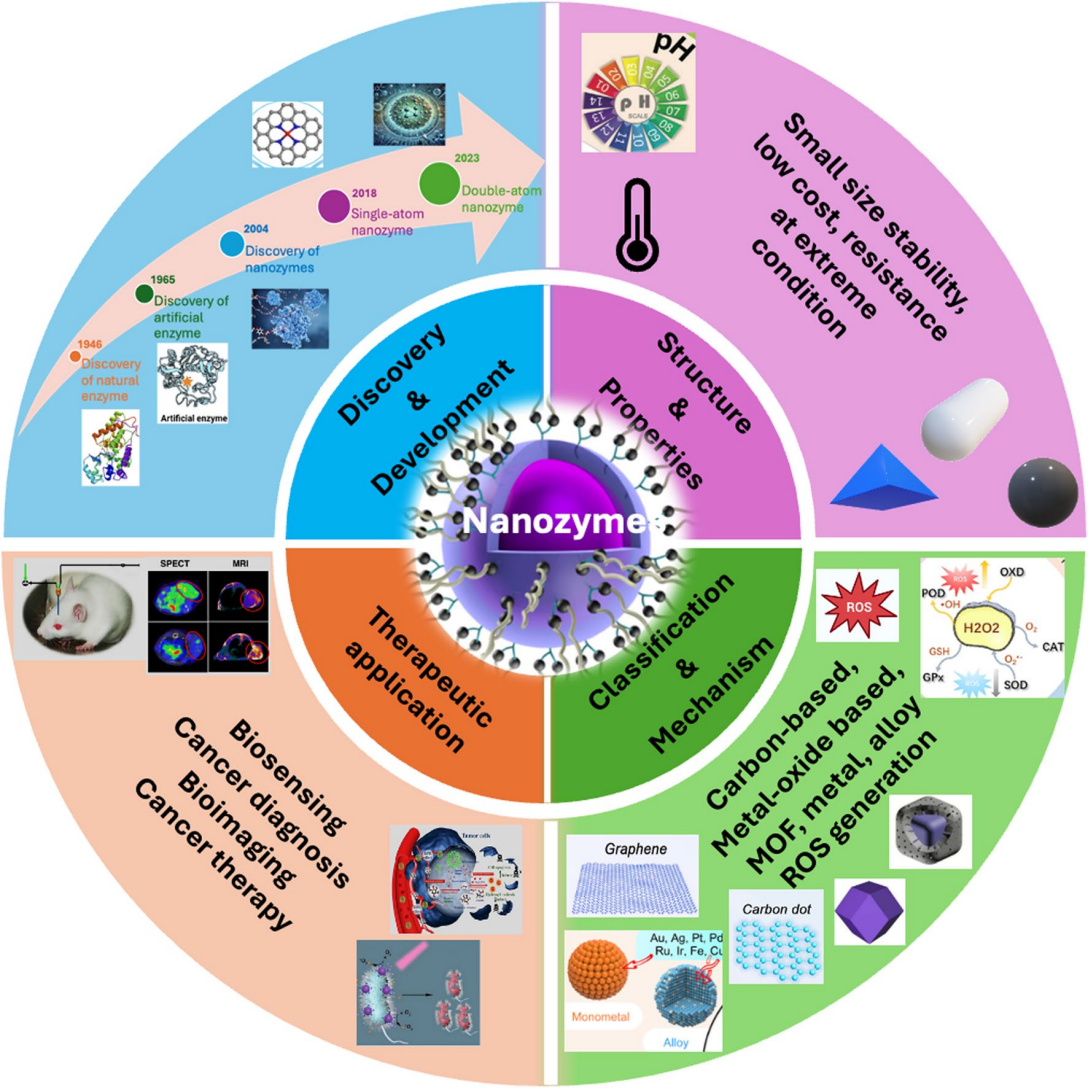
Overview of nanozyme discovery, properties, classification, and therapeutic applications

The physicochemical properties of nanozymes play a pivotal role in determining their catalytic activity, stability, and overall effectiveness in biomedical applications. These properties include size, shape, surface charge, and composition, all of which significantly influence the interaction of nanozymes with biological systems [33]. For example, the size of nanozymes, typically ranging from 1 to 100 nm, affects their surface-to-volume ratio, which in turn impacts their catalytic efficiency and reactivity. Smaller nanozymes often exhibit enhanced catalytic activity due to the greater number of active sites available for reactions [2]. Nanozyme shape can also dictate their performance; for example, spherical nanoparticles may be better able to penetrate into cells than larger or irregularly shaped particles. Surface charge is another critical factor, as it influences the stability of nanozymes in biological fluids and their interactions with cellular membranes. Additionally, the material composition of nanozymes, whether they are metal-based [7], metal oxide [34], or carbon-based [35, 36], determines their intrinsic catalytic properties and the types of reactions they can catalyze. By carefully tailoring these physicochemical properties, researchers can optimize nanozymes for specific applications in cancer therapy and diagnostics, enhancing their efficacy and safety in clinical settings. Figure 3 provides an overview of the properties, classification, and therapeutic applications of nanozymes.

## Nanozyme classification

Nanozymes can be categorized into two distinct groups based on their properties and functions. In terms of their properties, they are divided into two primary categories: carbon-based nanozymes and metal-based nanozymes. Each group demonstrates specific characteristics and applications that leverage their intrinsic properties for various biochemical processes [37].

### Based on their properties and composition

#### Carbon-based

Carbon-based nanozymes are classified into four primary dimensions: zero-dimensional (0D), one-dimensional (1D), two-dimensional (2D) [38], and three-dimensional (3D) nanozymes. Zero-dimensional carbon-based nanozymes, such as carbon dots and fullerenes, are spherical nanoparticles with excellent optical properties and biocompatibility and are suitable for bioimaging and drug delivery. One-dimensional carbon-based nanozymes, such as carbon nanotubes, have a cylindrical structure with a high aspect ratio and electrical conductivity, enabling effective biosensing and targeted drug delivery. Two-dimensional carbon-based nanozymes, such as graphene and graphene oxide, exhibit remarkable mechanical strength, high surface area, and excellent electron mobility, making them ideal for catalysis, energy storage, and enzyme immobilization. Classifying carbon-based nanozymes based on their dimensions can aid researchers in understanding their distinct properties and in designing them for specific applications in nanomedicine and environmental remediation.

#### Carbon-based

**Fig. 4:**
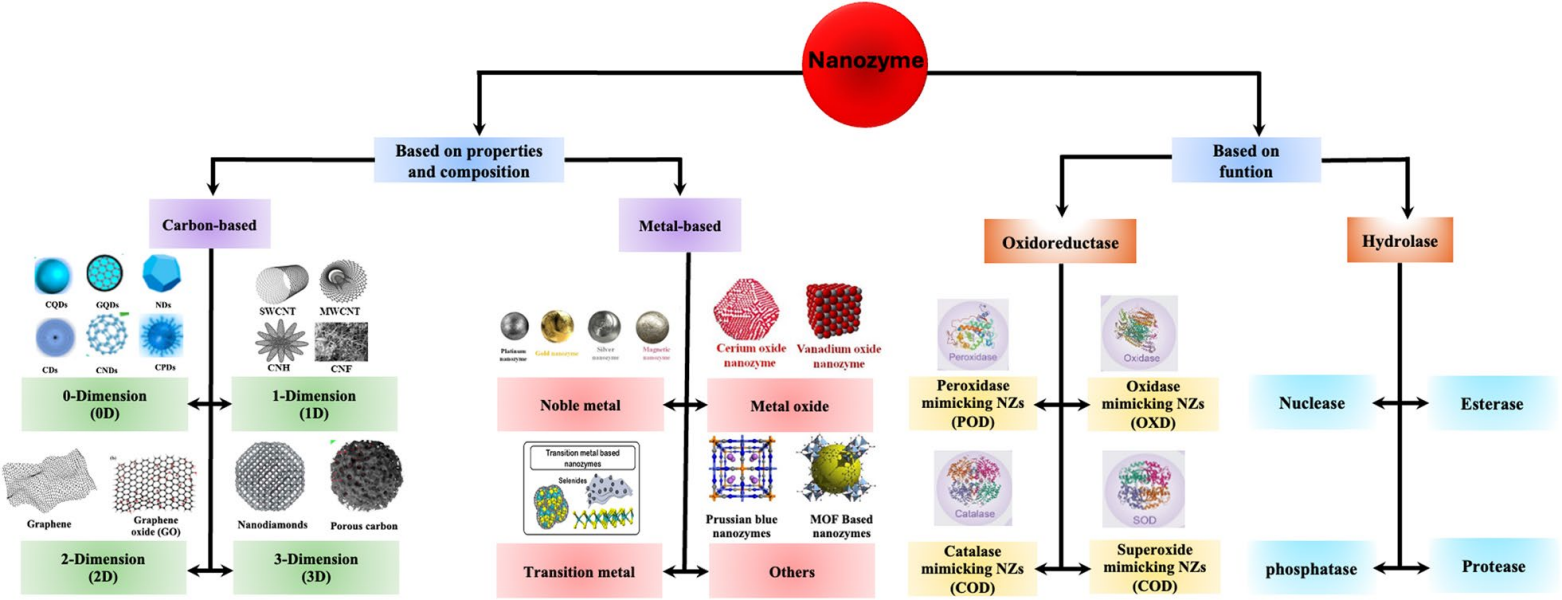
Classification of nanozymes: a comprehensive overview based on properties and functions [20]

Metal-based nanozymes can be categorized into four subgroups: noble metals [39, 40], transition metals [41], metal oxides [34], and others [42]. Metal-based nanozymes, composed of metals such as gold, silver, platinum, and iron oxide, mimic natural enzymes and exhibit unique properties such as a high surface area, tunable catalytic activity, and stability. Their enzyme-like functions facilitate biochemical reactions, making them valuable for targeted drug delivery, photothermal therapy, and biosensing [37]. Functionalization with targeting ligands enhances their specificity for cancer cells, minimizing damage to healthy tissues. The high biocompatibility and low toxicity of these materials make them suitable for in vivo applications. Future developments in metal-based nanozymes are expected to advance cancer treatment strategies and improve diagnostic techniques. A detailed classification of nanozymes is summarized in Fig. 4.

### Based on their function

**Fig. 5:**
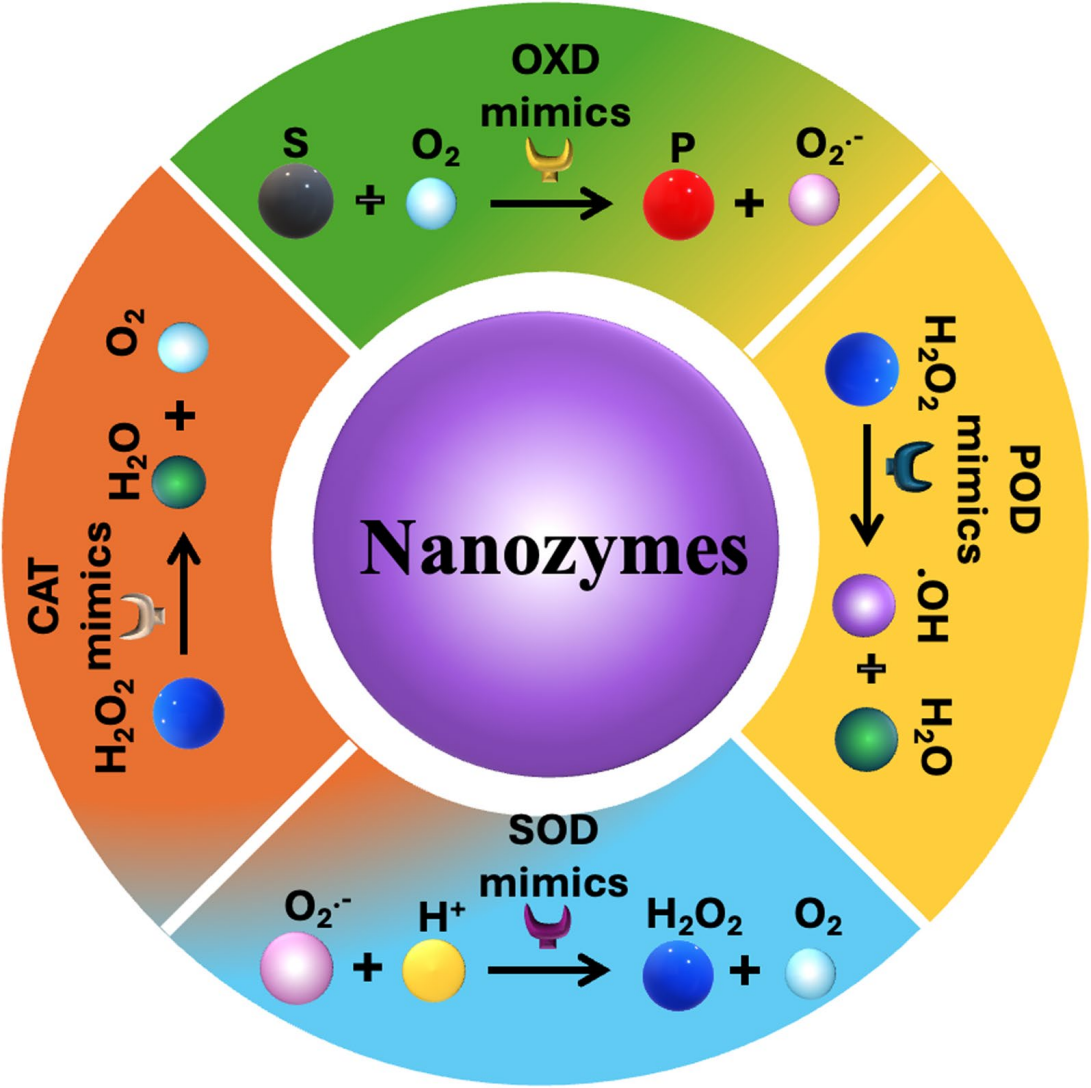
Schematic representation showing the catalytic reactions of different oxidoreductases and their mimics, including OXD, POD, SOD, and CAT

Based on their catalytic activities, nanozymes can be classified into two main groups: oxidoreductase and hydrolase. The oxidoreductase is further classified into four subgroups, including oxidase-like (OXD), peroxidase-like (POD), catalase-like (CAT), and superoxide dismutase-like (SOD) activities [24, 43–45]. Since almost all nanozymes reported for cancer theranostics possess oxidoreductase-like activities, we mainly focus on oxidoreductase nanozymes in this section. This classification framework helps explain the unique properties and potential applications of nanozymes in biomedicine and catalysis. The catalytic reaction mechanism and classification of oxidoreductases are shown in detail in Fig. 5.

#### POD-like nanozymes

Peroxidase, a naturally occurring enzyme, is found in a diverse range of organisms, including plants, humans, and bacteria [46]. These enzymes catalyze oxidation‒reduction reactions. They specifically facilitate the breakdown of hydrogen peroxide (H_2_O_2_), which produces water and free radicals, and H_2_O_2_ is a toxic byproduct formed during the respiration of oxygen [47]. The general mechanism of action of peroxidase mimetics involves the generation of reactive oxygen species (ROS) through mechanisms such as the Haber–Weiss reaction and the Fenton reaction [19]. These nanozymes, which are designed to mimic the enzymatic activity of natural peroxidases, can enhance the therapeutic efficacy of existing treatments by increasing local ROS levels within tumors, thereby promoting apoptosis and inhibiting tumor growth. The reaction pathway of POD-like nanozymes is shown in Eqs. (1) and (2).

POD-like nanozymes also feature unique properties, such as stability, ease of preparation, tunable activity, and functionalization for targeted delivery, make them suitable candidates for combination therapies [48]. Leveraging the advantages of nanozymes, researchers aim to develop more effective and less toxic cancer treatment strategies, paving the way for personalized therapeutic approaches in oncology. POD-like nanozymes have been extensively studied for their potential applications in biosensing [31], environmental remediation [49], and therapeutic interventions [50]. Peroxidase has been widely used in various industries, including pharmaceuticals and health, food and agriculture, paper and printing, and chemicals. However, Like proteins, peroxidase enzymes are susceptible to environmental factors, including temperature, pH, and ionic strength, which makes their storage challenging [51].12

#### OXD-like nanozymes

Oxidases facilitate the oxidation of a substrate, acting as an electron donor, and convert it into its oxidized form using oxygen as the electron acceptor under standard conditions [52]. This reaction results in the generation of either water (H_2_O) or hydrogen peroxide (H_2_O_2_). In contrast to peroxidases, oxidases do not require hydrogen peroxide as a reactant. Instead, they generate H_2_O_2_ and, in certain instances, superoxide radicals [53]. Consequently, oxidase and oxidase-like nanozymes can effectively transform colorless substrates into colorful products. Oxidase-mimicking (OXD-like) nanozymes represent a class of synthetic enzymes that replicate the functionality of natural oxidases. These nanozymes catalyze substrate oxidation by facilitating electron transfer to molecular oxygen (O₂), generating reactive oxygen species (ROS) or other oxidized products. Importantly, OXD-like nanozymes support redox reactions, making them valuable for various applications. With the rapid development of nanozyme technologies, oxidase-like nanomaterials have emerged as viable substitutes for natural oxidases, offering adjustable catalytic properties and enhanced stability. Researchers are increasingly focusing on these nanozymes, especially those that target specific substrates, including glucose, cysteine, and cytochrome *c*. [54]. Oxidase mimics (OXDs) have emerged as promising candidates in various fields, including biocatalysis, enzyme development, analytical chemistry, and sensing [55].34

#### CAT‑like nanozymes

Catalase-mimicking (CAT-like) nanozymes are synthetic enzymes designed to replicate the functions of natural catalases. These nanozymes effectively catalyze the breakdown of hydrogen peroxide (H_2_O_2_) into water (H_2_O) and oxygen (O_2_), helping to reduce oxidative stress and safeguarding biological systems from the damaging effects of reactive oxygen species (ROS). CAT-like nanozymes are characterized by their high intrinsic activity and cost-effectiveness, positioning them as promising alternatives to natural enzymes in various biomedical applications [48, 56]. Although they are generally considered biologically inert, nanoscale metallic materials have been shown to exhibit intrinsic enzymatic characteristics akin to those of natural enzymes due to their unique structures and electronic properties [57]. The combination of affordability, biocompatibility, ease of synthesis, and activity control has led to the widespread expectation that these metal nanomaterials will replace natural CAT in numerous application domains [58, 59]. Consequently, in recent years metal-based CAT-like nanozymes have attracted significant attention, particularly in the form of gold (Au), silver (Ag), platinum (Pt), and palladium (Pd) nanoparticles (NPs) and their nanocomposites.

#### SOD‑like nanozymes

Superoxide dismutase (SOD)-like nanozymes are artificial enzymes that mimic the activity of natural superoxide dismutase. These nanozymes catalyze the dismutation of superoxide radicals (O_2_^•–^) into hydrogen peroxide (H₂O₂) and molecular oxygen (O₂), which play crucial roles in protecting biological systems from oxidative stress by neutralizing harmful superoxide radicals. Natural superoxide dismutases (SODs), which generally consist of proteins and metal cofactors, are crucial for the survival of aerobic cells. They are present in most prokaryotic organisms and some eukaryotic cells and are widely distributed across different organelles [11]. On the basis of the cofactors used, natural SOD enzymes are classified into four types: copper-zinc SOD (CuZnSOD), manganese SOD (MnSOD), iron SOD (FeSOD), and nickel SOD (NiSOD) [60]. SOD mimetics are integral to numerous redox-active functions, such as neutralizing reactive oxygen species, serving as anti-inflammatory and antioxidative agents, and enhancing stem cell proliferation. Table 1 presents comprehensive classification of nanozymes categorized based on their catalytic activity.

**Table 1:** Comparison of common catalytic mechanisms involved in nanozyme activity

Activities	Mechanisms	Specific Functions	References
POD	ROS generation	Facilitating ROS generation through specific reactions (such as Fenton and Haber–Weiss reactions), which may involve contributions from both substrates and nanozymes	[6, 68–75]
	Electron transfer	Enhancing and transferring electrons without generating •OH radicals, or even suppressing •OH radical formation	[2, 76, 77]
OXD	Reactive species generation	Generating free radicals or ROS by activating either substrates or dissolved oxygen	[78–83]
	Electron transfer	Enhancing electron enrichment and facilitating electron transfer through redox reactions	[84, 85]
CAT	Adsorption activation	Facilitating H_2_O_2_ decomposition due to high adsorption energy	[86]
	Redox reaction	Decomposing H_2_O_2_ by redox reaction	[75, 87, 88]
SOD	Adsorption activation	Enhancing disproportionation through adsorption	[89, 90]
	Electron transfer	Achieving disproportionation through free radical or redox reactions	[88, 91, 92]

Table content reprinted from [13]

56

### Others

Hydrolase nanozymes are a type of artificial enzyme that mimics the catalytic activity of natural hydrolases, which facilitate the hydrolysis of chemical bonds in various substrates, such as proteins, lipids, and nucleic acids [61–64]. These nanozymes can be categorized based on their specific catalytic functions, including esterase, protease, and phosphatase functions [65, 66]. In cancer therapy, hydrolase nanozymes exhibit significant promise due to their ability to selectively target and degrade tumor-associated biomolecules, thereby disrupting the proliferation and survival of cancer cells. For instance, esterase-like nanozymes can hydrolyze ester bonds in prodrugs, transforming them into active therapeutic agents that effectively eliminate cancer cells. Protease-mimicking nanozymes can also facilitate the breakdown of extracellular matrix components, thereby enhancing penetration of tumor tissue precedes drug delivery. The adaptability and tunability of hydrolase nanozymes render them indispensable tools in developing targeted cancer therapies, thereby augmenting treatment efficacy while minimizing the side effects commonly associated with conventional therapies [67]. Their functionality under physiological conditions further augments their potential for clinical applications in cancer treatment.

## Application of nanozyme in cancer treatments

### Single therapy

#### Chemotherapy

**Fig. 6:**
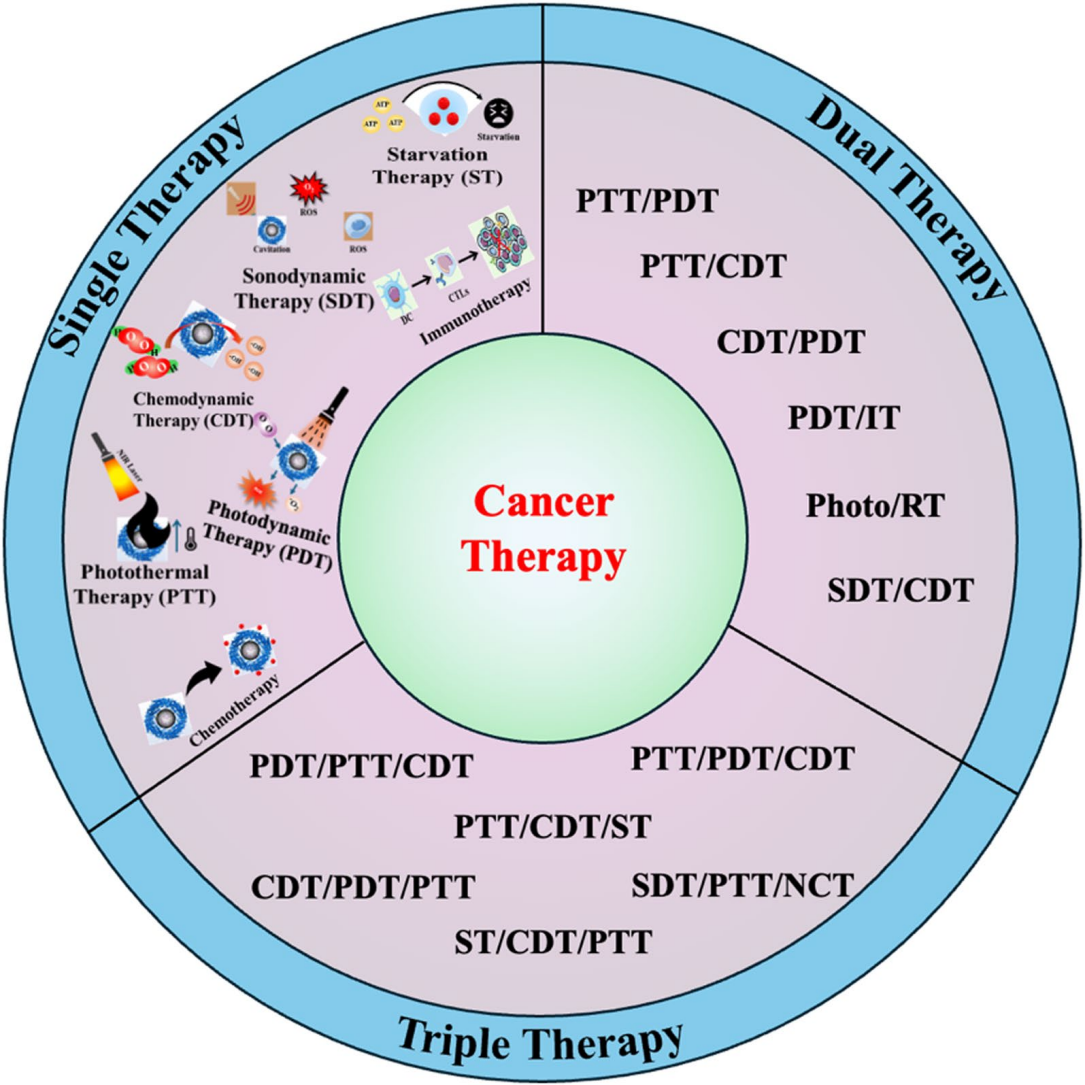
Application of nanozyme in cancer therapy

Cancer chemotherapy uses chemicals with cytostatic and cytotoxic effects to inhibit tumor progression and destroy cancerous cells [93, 94]. While traditional chemotherapy can be effective, it is well known for causing severe side effects, primarily due to the nonselective absorption of these drugs by both healthy and cancerous cells in tissues and organs [95]. At present, a variety of pharmaceuticals are employed in cancer chemotherapy. However, the unique characteristics of the tumor microenvironment (TME), including conditions of hypoxia and elevated levels of hydrogen peroxide (H_2_O_2_), significantly hinder the therapeutic efficacy of these chemotherapeutic agents [96, 97]. Nanozymes have emerged as a transformative tool in chemotherapy and cancer treatments due to their unique enzyme-like properties and ability to enhance therapeutic efficacy. These engineered nanomaterials can mimic natural enzymes, facilitating the generation of reactive oxygen species (ROS) that induce oxidative stress and promote cancer cell death. In chemotherapy, nanozymes can serve as drug delivery systems, improving the solubility and bioavailability of chemotherapeutic agents while minimizing systemic toxicity. Nanozymes offer several advantages over traditional mimetic enzymes, including enhanced efficiency and the ability to function in human health applications. They are stable under extreme conditions and can be produced at scale, making them a cost-effective choice for industrial and medical uses. They can also enhance the effectiveness of existing treatments by overcoming drug resistance mechanisms, enabling more targeted and efficient tumor ablation. When combined with other therapeutic modalities such as Photodynamic Therapy (PDT), Photothermal Therapy (PTT), Photoacoustic Therapy (PAT), Chemodynamic Therapy (CDT), and Sonodynamic Therapy (SDT), this approach is referred to as chemo-synergetic therapy. Chemo-synergetic therapy improves the overall effectiveness of cancer treatment [15, 98]. With its accessibility and ongoing research aimed at improving efficacy and reducing side effects, chemotherapy remains a key, cost-effective option in the fight against cancer. Figure 6 provides a detailed illustration of different types of cancer therapy strategies.

#### Photothermal therapy (PTT)

**Fig. 7:**
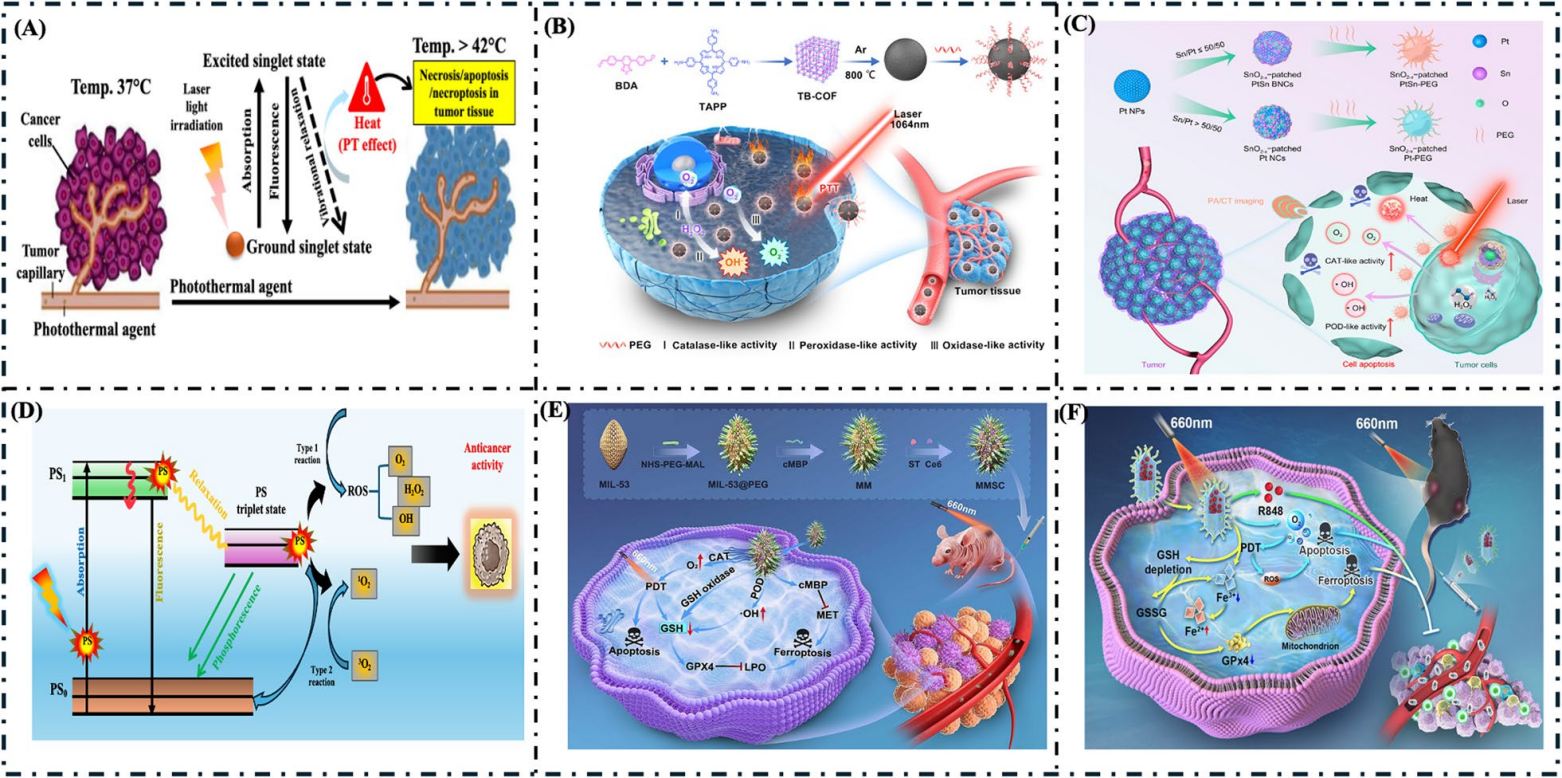
**A** Mechanism of NIR photothermal therapy. **B** Schematic illustration of the COF-derived N-doped carbon nanozyme with multiple enzyme-like activities and its anticancer mechanism, reprinted with permission from [103]. Copyright 2023, American Chemical Society. **C** Schematic illustration of PtSn BNCs for the phototheranostic effect and photothermal-enhanced catalytic therapy. Reprinted with permission from [104]. Copyright 2023, American Chemical Society. **D** Jablonski diagram illustrating the mechanism of photodynamic therapy (PDT). **E** Schematic diagram illustrating the synthesis of MMSC and its mechanism to amplify ferroptosis via MET inhibition and photodynamic therapy, reprinted with permission from [117]. Copyright 2024, Elsevier Ltd. **F** (a) Schematic illustration of synthesis tactics for Fe-TCPP-R848-PEG (Fe-MOF-RP) and (b) mechanism of light-driven nanozymes against tumor therapy reprinted with permission from [118]. Copyright 2023, American Chemical Society

Photothermal Therapy (PTT) is an innovative cancer treatment modality that uses light-absorbing nanoparticles to convert light energy into heat, specifically targeting and destroying cancer cells. Using photothermal agents (PTAs) that convert near-infrared (NIR) light into thermal energy, PTT induces localized heating, causing cellular damage or death while preserving healthy surrounding tissues [99, 100]. Figure 7A illustrates the mechanism of photothermal therapy. PTT is particularly advantageous because of its noninvasive nature, precise spatial control, and ability to be combined with other therapeutic approaches, such as chemotherapy and immune therapy, enhancing overall treatment efficacy [101, 102]. As research advances, photothermal therapy holds significant promise for improving cancer treatment outcomes and expanding therapeutic options in various medical fields. Nanozymes can significantly enhance PTT efficacy by serving as efficient PTA that converts light energy into heat to selectively destroy cancer cells. By integrating nanozymes into PTT, researchers can improve treatment specificity, reduce the severity of side effects, and potentially overcome challenges associated with conventional therapies, making them a promising tool in the fight against cancer. Overall, PTT represents a promising alternative or complement to conventional cancer therapies, offering a more targeted and less toxic approach to tumor management.

Wan et al. presented a successful paradigm for developing an N-doped carbon-based nanozyme (CN-PEG) for targeted tumor catalytic therapy and a second near-infrared (NIR-II) PTT using an N-containing COF as the precursor [103]. The as-prepared CN-PEG was found to exhibit three enzyme-like activities: (i) oxidase (OXD)-like activity to induce adequate O_2_^•–^ in the presence of O_2_; (ii) catalase (CAT)-like activity to catalyze the disproportionation of H_2_O_2_ and produce O_2_ and H_2_O; (iii) peroxidase (POD)-like activity to reduce H_2_O_2_ and generate •OH. Taking advantage of these three enzyme activities and the excessive level of H_2_O_2_ in tumor tissue, sufficient ROS can be generated to effectively kill cancer cells, thereby realizing catalytic therapy for cancer. Furthermore, the nanozyme exhibits a strong NIR-II light absorption capacity to transform light energy into thermal energy with a high photothermal conversion capability to kill cancer cells. Both in vitro and in vivo experiments demonstrate that this “all-round” nanozyme enables synergistic enhanced catalytic therapy and NIR-II photothermal therapy of cancer, as illustrated in Fig. 7B. The nanozyme also exhibits selective killing efficiency toward cancer cells due to the difference in the expression level of H_2_O_2_ between tumor and normal tissue and the spatiotemporal controllability of laser irradiation.

Similarly, Zhu et al. developed dual nanozyme-driven PtSn bimetallic nanoclusters for metal-enhanced tumor PTT and catalytic therapy [104]. The PtSn bimetallic nanoclusters (BNCs) with adjustable compositions by varying the amount of Sn precursor used. This exploration of composition-dependent structural evolution and activities is presented in Fig. 7C. Among the investigated BNCs, the Pt_50_Sn_50_ BNCs exhibited the highest enzymatic catalysis and photothermal properties. Notably, the catalytic activities of the Pt_50_Sn_50_ BNCs could be significantly enhanced under the influence of NIR laser irradiation. Multicellular tumor spheroid-related experiments and in vivo results demonstrated the exceptional penetration behavior and enhanced catalytic-anticancer efficacy of Pt_50_Sn_50_ BNCs. Furthermore, the coexistence of Pt and Sn in the Pt50Sn50 BNCs enabled the detection of Pt_50_Sn_50_ BNCs using positron emission tomography (PET) and computed tomography (CT) imaging, facilitating the monitoring of their in vivo fate within the tumor.

#### Photodynamic therapy (PDT)

Photodynamic therapy (PDT) is a minimally invasive cancer treatment method that uses the synergistic effects of light, a photosensitizer, and oxygen to induce cytotoxic effects in tumor cells [105, 106]. The mechanism involves the administration of a photosensitizer, a light-sensitive compound that preferentially accumulates in malignant tissues [107, 108]. Upon exposure to specific wavelengths of light, typically within the visible to near-infrared spectrum, the photosensitizer activates and transitions to a higher energy state [109]. This activated state facilitates the transfer of energy to ground-state oxygen molecules in the tissue, generating reactive oxygen species (ROS), such as singlet oxygen [110, 111]. These ROS are highly reactive and can cause substantial damage to cellular components, including lipids, proteins, and DNA, ultimately leading to cell death through apoptosis or necrosis [112]. The detailed mechanism of PDT is illustrated in Fig. 7D. The efficacy of PDT is contingent upon various factors, including the selection of the photosensitizer, the light dose intensity, and the oxygen availability within the tumor microenvironment. Consequently, PDT has emerged as a versatile and targeted modality for treating diverse cancers while minimizing damage to surrounding healthy tissues [113, 114]. The application of PDT using nanozymes represents a promising strategy in cancer therapy, leveraging the unique properties of nanozymes to enhance ROS generation, improve targeting, and provide a dual mechanism of action [115, 116]. Ongoing research is focused on optimizing nanozyme formulations and treatment protocols to maximize their therapeutic potential and improve patient outcomes in clinical settings.

Xu et al. reported that an enzyme-based drug delivery system could amplify ferroptosis via MET inhibition and PTT [117]. A multifunctional enzyme-based nano complex named MIL-53@cMBP@ST/Ce6 (MMSC) was synthesized to enhance cancer therapy. The MMSC nanocomplex was engineered by modifying the cMBP peptide on the surface of MIL-53 and encapsulating ST and Ce6 (Fig. 7E). In vitro studies demonstrated the ability of MMSC to attenuate MET signaling pathways, induce intracellular lipid peroxidation, and promote ferroptosis. Furthermore, transcriptomic analyses revealed significant modulation of ferroptosis-related genes in laser irradiation-treated cells, and Kyoto Encyclopedia of Genes and Genomes (KEGG) pathway analysis highlighted the involvement of MET signaling pathways and GSH metabolism pathways in ferroptosis. A novel strategy was demonstrated by Xu et al. and coworkers for the mechanistic investigation of augmented ferroptosis in tumor therapy, highlighting the potential of MMSC as a versatile and potent anti-tumor nanoplatform.

Fan et al. reported a light-triggered nanozyme Fe-TCPP-R848-PEG (Fe-MOF-RP) designed to remodel the immunosuppressive microenvironment [118]. This study presents a self-oxygenating photodynamic system using Fe-TCPP metal–organic frameworks (Fe-MOFs) to enhance cancer photoimmunotherapy via ferroptosis (see Fig. 7F). The PEG-coated Fe-MOFs exhibit excellent histocompatibility and tumor-targeting properties, which allow for prolonged retention of therapeutic agents in circulation and increased accumulation in tumor tissues. At the tumor site, Fe-MOFs facilitate the breakdown of hydrogen peroxide, resulting in oxygen release that alleviates tumor hypoxia. The system also responds to the tumor microenvironment, releasing high concentrations of TCPP and Fe^2+^ in the presence of elevated glutathione (GSH) levels. The TCPP-mediated photodynamic therapy and the iron-driven Fenton reaction induce ferroptosis in tumor cells. R848 was included to enhance the immune response against tumor-associated antigens (TAAs), contributing to sustained and targeted antitumor immunity development.

#### Chemodynamic therapy (CDT)

**Fig. 8:**
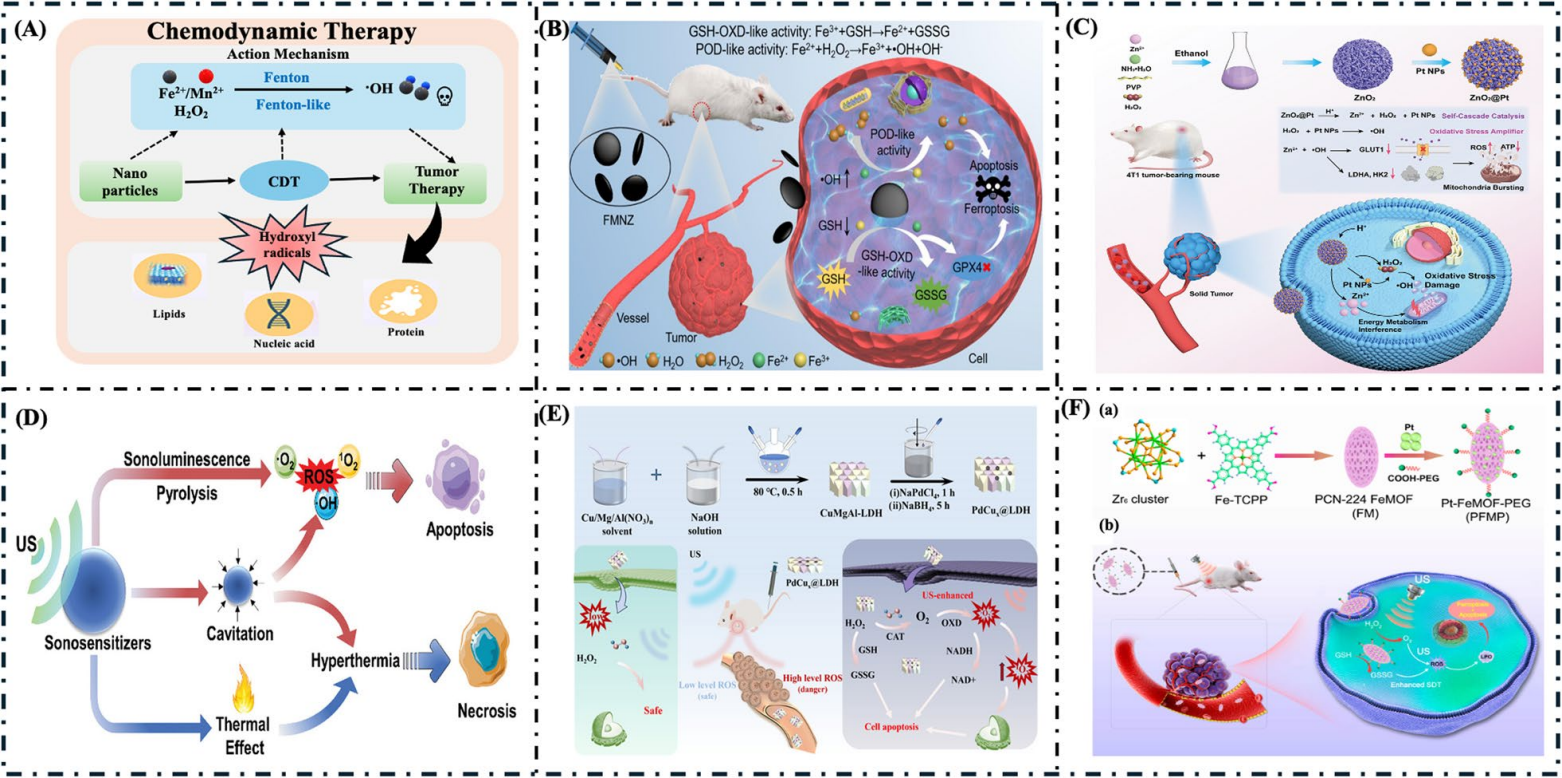
**A** Possible mechanism of sonodynamic therapy (SDT). **B** Schematic illustration of self-cascading MNZs with dual enzymatic GSH-OXD-like and POD-like activities for tumor-specific CDT, reprinted with permission [123]. Copyright 2024, Elsevier Ltd. **C** Schematic illustration of H_2_O_2_ self-suppling synergistic energy metabolism interference enhanced catalytic therapy, reprinted with permission [124]. Copyright 2024, Wiley–VCH GmBH. **D** Schematic diagram of potential mechanisms of SDT, reprinted with permission [135]. Copyright 2024, Wiley–VCH GmBH. **E** Schematic diagram for preparing the PdCu_*x*_@LDH allays nanozymes and the proposed antitumor mechanism, reprinted with permission from [133]. Copyright 2024, American Chemical Society. **F** Schematic representation of the (a) synthesis of the PFMP nanozyme and (b) tumor accumulation and tumor microenvironment-responsive enhanced SDT mechanism, reprinted with permission from [134]. Copyright 2024, American Chemical Society

Chemodynamic therapy (CDT), a novel cancer treatment, uses Fenton-like reactions involving various metal elements to generate ROS to eliminate tumor cells while selectively minimizing adverse effects [119]. This treatment modality is particularly advantageous due to its capacity to specifically target cancer cells while minimizing adverse effects on surrounding healthy tissues. CDT’s advantages include tumor selectivity, endogenous initiation, hypoxia modulation, and cost-effectiveness. The Fenton reaction, introduced by Fenton in 1894, uses iron and hydrogen peroxide to produce harmful hydroxyl radicals (•OH) that break down organic pollutants [120]. This reaction, first described in 1894, is now widely recognized for its role in degrading contaminants [121]. Recently, many researchers have sought to apply Fenton reactions and similar processes to broader fields. Clearly, the swift advancement of nanomaterials is crucial for effectively applying Fenton/Fenton-like reactions in the biomedical field. Integrating nanozymes into CDT boosts its effectiveness by enhancing reactive ROS production, enabling targeted delivery, and allowing for potential combination therapies. Current research aims to optimize chemodynamic agents and treatment protocols to maximize therapeutic benefits and improve patient outcomes in clinical practice. Figure 8A illustrates the detailed mechanism of CDT [122].

Qian et al. reported the structural engineering of magnetite nanozymes for enhanced chemodynamic cancer therapy [123]. Three multifunctional nanomaterialsMNZ forms, flaky, elliptic, and spherical, were developed with dual enzymatic functions: GSH oxidation and H_2_O_2_ decomposition. These functions reduce intracellular OH consumption and generate OH while depleting GSH. The authors demonstrated that these self-cascading MNZs exhibit shape-dependent cytotoxic efficacy at both the cellular and tissue levels. Notably, FMNZ exhibited a remarkable ability to shrink tumors in an aggressive triple-negative breast cancer model. Figure 8B shows the schematic illustration of self-cascade MNZs with dual enzymatic GSH-OXD-like and POD-like activities for tumor-specific CDT.

Sun et al. reported that Zinc-Based ROS amplifiers trigger cancer Chemodynamic/ion interference therapy through self-cascade catalysis [124]. In this study, a zinc-based nanozyme platform was designed for the self-supply of H_2_O_2_ and to mitigate ion interference through self-cascade catalysis (Fig. 8C). ZnO_2_@Pt, a self-cascading nanozyme, enhances oxidative stress in tumors through a combination of catalytic and ion-interference therapies. This approach, which maximizes the anti-tumor effect, addresses the limitations of conventional catalytic therapy.

#### Sonodynamic therapy (SDT)

Sonodynamic therapy (SDT) is a novel cancer treatment modality that uses low-intensity ultrasound waves to activate sonosensitizers, generating reactive oxygen species (ROS) that can induce cancer cell death [125, 126]. In contrast to conventional cancer phototherapy, which is limited by inadequate penetration at the tumor site, SDT is characterized by a greater penetration depth arising from the ultrasonic cavitation effect [127]. This mechanism not only undermines the integrity of tumor blood vessels but also selectively ablates tumor cells, all while minimizing damage to adjacent healthy tissues [128–130]. Using ultrasound enables deeper tissue penetration and precise targeting of tumors, thereby minimizing damage to surrounding healthy tissues. SDT has demonstrated efficacy in treating various types of cancers, particularly those that are challenging to access or resistant to conventional therapies. Based on the mechanism of SDT-mediated cell death, the choice of sonosensitizer for cancer therapy has become an essential aspect of current investigations. SDT, combined with other treatment modalities such as chemotherapy, PDT, PTT, and immunotherapy, has been found to achieve a synergistic therapeutic effect for tumors in vitro and in vivo [130]. The application of nanozymes in SDT represents a significant advancement in enhancing the efficacy of this treatment approach. Ongoing research is also exploring using SDT and imaging techniques to allow for real-time monitoring of treatment response, further improving its clinical applicability. As the understanding of sonosensitizers and ultrasound parameters has advanced, sonodynamic therapy has become a valuable tool in the fight against cancer, offering a noninvasive and targeted approach to tumor ablation [131, 132]. Figure 8D illustrates the detailed mechanism of the SDT.

Mo et al. pioneered a groundbreaking approach that developed a layered double hydroxide-based PdCux@LDH alloy nanozyme designed for singlet oxygen-boosted sonodynamic therapy (SDT) [133]. This innovative redox nanozyme showcases surface-dependent multienzyme-mimicking activities combined with exceptional ultrasound sensitivity, making it a powerful agent for tumor-specific therapy. By leveraging synergistically enhanced cascade catalytic reactions, the PdCux@LDH alloy nanozyme efficiently generates singlet oxygen (^1^O_2_) to target cancer cells (Fig. 8E). The researchers systematically explored the influence of metal ratios (x = 0.4 to 1.2) on enzyme-like activities, finding that PdCu0.8@LDH exhibited the highest catalytic efficiency. This is attributed to the synergistic electron transfer between Pd and Cu, which amplifies active sites and boosts oxidase (OXD)-like activity. Notably, the alloy nanozyme exposes dual catalytic active centers-Pd(111) and Pd(100) crystal planes—simultaneously emulating the functions of catalase (CAT) and OXD. Moreover, PdCux@LDH exhibited the ability to deplete overexpressed glutathione (GSH) through a redox reaction (Cu^2^⁺ + GSH → Cu⁺ + GSSG), effectively mitigating nontherapeutic ROS consumption caused by the presence of GSH. Ultrasound stimulation further amplified the nanozyme’s multienzyme-like activities, significantly enhancing reactive oxygen species (ROS) generation and ensuring robust therapeutic performance. This work marks a significant advancement in the field of SDT, offering a multifunctional platform for precise and effective cancer treatment.

Qian et al. developed a cutting-edge cascade nanozyme featuring platinum-coated porphyrin metal–organic frameworks (Pt@MOFs) engineered to amplify reactive oxygen species (ROS) production for enhanced sonodynamic theranostics of tumors. Under the influence of ultrasound (US), the MOF activates and produces reactive oxygen species (ROS) caused via sonosensitizer TCPP. Additionally, Pt acts as a catalyst to convert intratumoral H_2_O_2_ into O_2_, effectively reducing tumor hypoxia and enhancing the efficacy of O_2_-dependent therapies. Within the tumor, the overexpression of glutathione (GSH) facilitates the reduction of ferric ions (Fe^3+^) to ferrous ions (Fe^2+^), thereby diminishing the ROS-scavenging capacity of GSH and promoting ROS toxicity. Furthermore, the autofluorescence of TCPP enables the PFMP nanozyme to track its internal circulation, allowing for precise stereotactic delivery. This innovative design seamlessly integrates catalytic precision with sonosensitizer efficiency, marking a significant leap forward in tumor-targeted therapy [134]. Figure 8F shows the schematic illustration of this work.

#### Immunotherapy

Immunotherapy is an innovative cancer treatment strategy that uses the body’s immune system to recognize and destroy cancer cells. Unlike traditional therapies that directly target tumors, immunotherapy aims to enhance or reestablish the natural ability of the immune system to fight cancer [136]. This can be achieved through various strategies, including the use of monoclonal antibodies, immune checkpoint inhibitors, cancer vaccines, and adoptive cell transfer therapies, such as CAR-T-cell therapy [137]. Immunotherapy has demonstrated remarkable efficacy in treating numerous cancer types, including melanoma, lung cancer, and hematological malignancies, often resulting in long-lasting responses and improved survival rates [138]. One of the primary applications of immunotherapy is its capacity to target tumors resistant to conventional treatments, offering new hope for patients with advanced or metastatic disease [139, 140]. Using nanozymes in immunotherapy represents an advanced strategy in cancer treatment, capitalizing on their unique properties to augment immune responses directed against tumors. By enhancing antigen presentation, activating immune cells, and modifying the tumor microenvironment, nanozymes can substantially improve the efficacy of immunotherapeutic approaches. Current research focuses on optimizing these nanozymes for clinical applications to enhance patient outcomes and expand the scope of immunotherapy in cancer treatment.

**Fig. 9:**
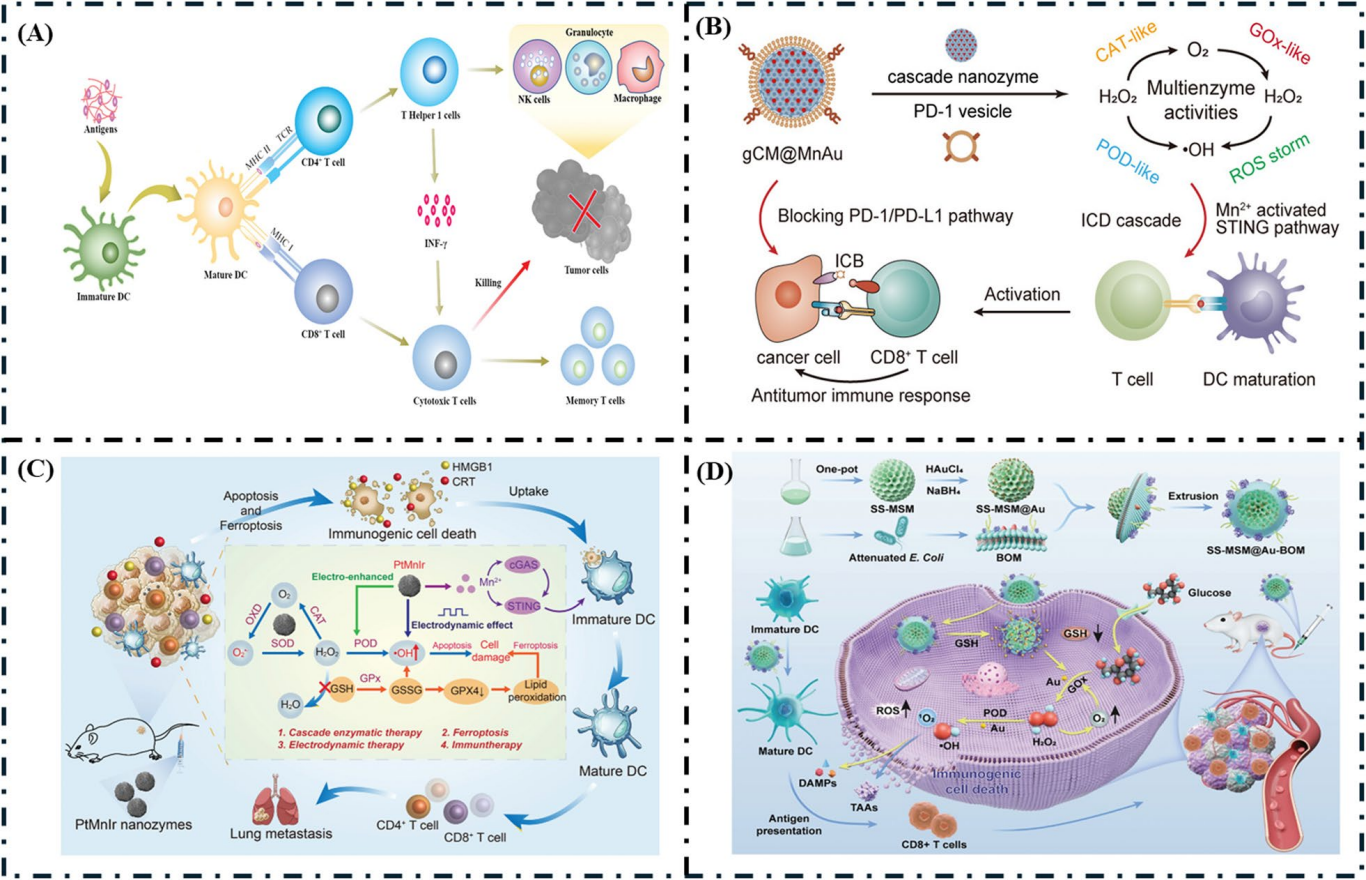
**A** Schematic illustration of the mechanism of cancer immunotherapy, reprinted with permission [147]. Copyright 2021, Wiley–VCH GmBH. **B** Schematic illustration of DC activation and PD-1/PD-L1 blockade mediated by gCM@mnau for cancer immunotherapy, reprinted with permission [141]. Copyright 2024, American Chemical Society. **C** Schematic diagram of the antitumor mechanism of PtMnIr nanozymes, reprinted with permission [142]. Copyright 2023, Wiley–VCH GmbH. **D** Schematic illustration of synthesis and tumor catalytic immunotherapy of SS-MSN@Au-BOM, (a) SS-MSN were prepared by the one-pot method, and Au nanozyme was grown on the surface of SS-MSN through NaBH_4_ reduction, reprinted with permission [143]. Copyright 2024, Wiley–VCH GmBH

Increasing overall treatment efficacy requires further research investigating the integration of immunotherapy with other modalities, such as chemotherapy, radiotherapy, and targeted therapies. As our understanding of the immune system and tumor microenvironment evolves, immunotherapy advances present promising avenues for personalized cancer treatment and enhanced patient outcomes. The detailed mechanism of immunotherapy is illustrated in Fig. 9A.

Interestingly, Zhang et al. designed genetically engineered cascading nanozymes for enhanced immunotherapy with ICB [141]. As depicted in Fig. 9B, gold (Au) nanoparticles with GOx-like activity were absorbed into manganese dioxide (MnO2) nanoparticles with CAT-like activity through electrostatic interactions to form the cores of MnAu nanozymes. These cores were coated with genetically engineered cell membranes (gCMs) overexpressing PD-1 receptors to obtain biomimetic nanozymes gCM@MnAu.These nanozymes induce an immune checkpoint blockade (ICB) cascade and stimulate STING activation, thereby maturing dendritic cells (DCs). Additionally, genetically engineered PD-1 vesicles block the PD-1/PD-L1 pathway. Core MnAu nanozymes augment the chimeric antigen receptor (CAR)-based immunotherapy (gCM-based ICB) by enhancing DC-mediated cross-priming of T cells. In turn, gCMs promote tumor accumulation through homologous targeting.

Li et al. developed “Spark” PtMnIr nanozymes for electrodynamic-boosted multienzyme tumor immunotherapy [142]. Figure 9C shows the schematic illustration of PtMnIr nanozymes. The PtMnIr nanozymes, synthesized through a one-pot hydrothermal method, exhibit multiple enzymatic activities and can induce ferroptosis in tumor cells through an “inner catalytic loop” mechanism. This nanodrug activates immune pathways and inhibits tumor metastasis, providing a synergistic approach to cancer therapy.

Zhang et al. developed a self-oxygenated biomimetic nanozyme for tumor catalytic immunotherapy [143]. As depicted in Fig. 9D(a). Mesoporous silicon nanoparticles (MSN) with disulfide bonds (SS-MSN) were synthesized using a one-pot method. Gold nanozymes (Au-Nanozyme) were then added to create SS-MSN-Au. To improve biocompatibility and enhance antitumor efficacy, SS-MSN-Au was combined with Bovine Ovarian Membrane (BOM) and processed through a liposome extrusion apparatus, resulting in SS-MSN-Au@BOM. GSH reduction triggers a cascade of enzyme activities that generate hydroxyl radicals and singlet oxygen, ultimately leading to the immunogenic death of cancer cells. The catalytic immunotherapy mechanism of SS-MSN-Au@BOM in vivo is illustrated in Fig. 9D(b).

Overall, this strategy suggests that the enzyme’s stability, tunability, and ease of production further enhance its potential as a valuable tool for developing innovative cancer immunotherapies. Moreover, advancements in immunotherapy, such as checkpoint inhibitors and CAR T-cell therapy, have shown promising results in improving survival rates and making it a vital component of modern cancer treatment.

#### Starvation therapy

Starvation therapy, also known as dietary restriction or caloric restriction, is a therapeutic approach that entails reducing caloric intake or limiting specific nutrients to achieve health benefits or treat certain medical conditions. This method is rooted in the notion that decreasing food consumption can induce metabolic alterations that facilitate cellular repair, enhance stress resistance, and contribute to overall well-being. In cancer treatment, starvation therapy aims to leverage the specific metabolic weaknesses of cancer cells, which usually depend on elevated levels of glucose and other nutrients for growth and proliferation. By limiting these nutrients, this therapy may slow tumor growth, enhance the efficacy of standard treatments like chemotherapy and radiation, and trigger apoptosis (programmed cell death) in cancer cells.

Lu et al. proposed a cascading catalysis system featuring the “core@paratroopers” structure, wherein hollow manganese dioxide (HMnO_2_) serves as “core” and ultra-small hybrid single-micelle (H-micelle) encapsulated with glucose oxidase (GOx) as “paratroopers” (H-micelle-GOx) [144]. The outer SiO_2_ layer of the H-micelle can effectively protect the GOx. Under hypoxic conditions, HMnO_2_ reacts with endogenous H_2_O_2_ to produce O_2_, thereby enhancing the catalytic efficiency of GOx for starvation therapy.

Chen et al. presented a pH/ROS dual-responsive enzyme-carrying nanoparticles for efficient starvation and oxidative therapy in cancer treatment [145]. The nanoparticles, composed of zeolitic imidazolate framework-8 (ZIF-8), glucose oxidase (GOx), and hyaluronic acid (HA), were meticulously designed to capitalize on the distinctive metabolic characteristics of cancer cells. GOx was covalently attached to HA to generate HA-GOx, exhibiting enhanced enzymatic activity and thermal stability compared to free Gox.

Cao et al. reported the demonstration of a cytochrome c oxidase-like nanozyme (copper–silver alloy nanoparticle, Cu-Ag NP) for nanocatalytic cancer therapy [146]. Loaded with bioreductive predrug (AQ4N), this Cu-Ag nanozyme exhibits unprecedented capabilities, enabling simultaneous starvation, ferroptosis, and chemical therapy with exceptional specificity. Notably, it can completely eliminate tumors and significantly prolong the survival rate of 4T1-tumor-bearing mice.

### Dual therapy

Combination cancer therapy strategies involve using multiple treatment modalities to enhance the overall cancer treatment efficacy, minimize the potential for resistance development, and improve patient outcomes. Single therapeutic strategies using nanozyme nanoplatforms have achieved positive treatment effects, demonstrating notable advantages in cancer therapy. However, several intrinsic drawbacks of single therapy are still unavoidable [148]. For example, although RT and chemotherapy have many applications and enhanced tumor eradication abilities, they suffer from severe side effects. CDT and starvation therapy possess the advantages of non-invasiveness and high selectivity but still face the shortcomings of H_2_O_2_ and O_2_ concentration dependence in the TME. Non-invasive and highly controlled phototherapy is limited by light penetration depth. Immunotherapy possesses broad applicability while exhibiting high inflammatory response and typical clinical reaction rates. Compared with the single therapy methods, integrating two or more therapy methods in one system for synergistic tumor therapy can achieve a “1 + 1 > 2” cumulative effect, reduced therapeutic agent dosage, and reduced side effects [8, 149]. Combination therapy generally exhibits better therapeutic effects than single therapy in preclinical and clinical studies and can break through the limitations of single therapy [150, 151]. This raises an exciting opportunity to integrate multiple therapy methods into one nanozyme-based nanoplatform for precision tumor therapy. Various combination therapies incorporating nanozyme-based nanoplatforms have recently been developed to achieve superior antitumor effects [41, 152, 153]. This section summarizes the recent representative nanozyme-based nanoplatforms in precision tumor therapy using bimodal therapy.

#### PTT/PDT and PDT/PTT

PTT/PDT combined cancer therapy refers to a synergistic treatment approach that integrates PTT and PDT to enhance treatment effectiveness. By combining PTT and PDT, this dual-modal therapy leverages the strengths of both techniques: PTT can strengthen the effectiveness of PDT by increasing the local temperature, thus improving the activation of the photosensitizer and promoting ROS generation [152]. Additionally, the heat generated by PTT can increase the permeability of tumor blood vessels, improving delivery of the photosensitizer to the tumor site. This combined approach targets cancer cells through multiple mechanisms and helps overcome potential resistance, ultimately leading to improved therapeutic outcomes and reduced side effects.

**Fig. 10:**
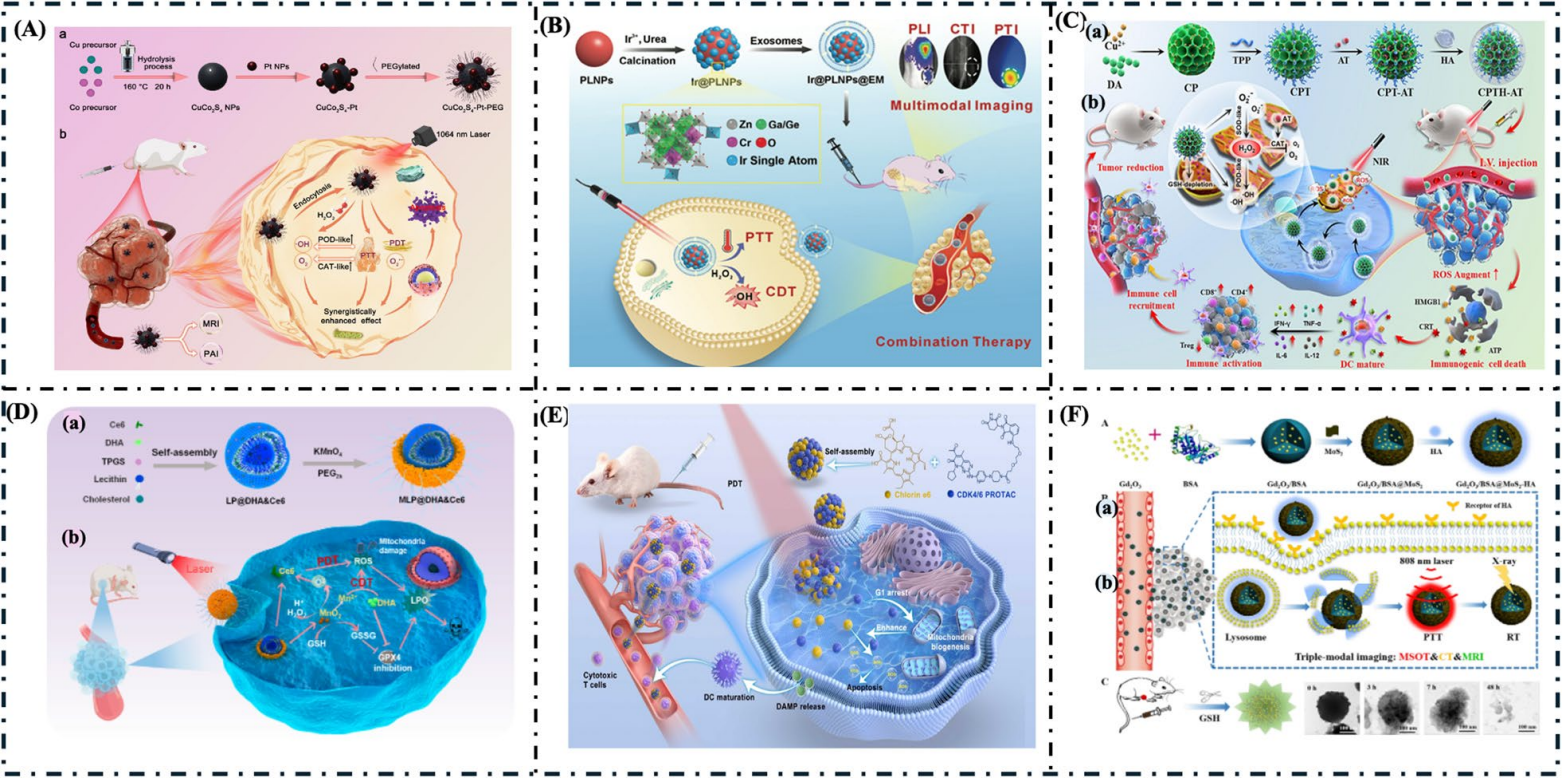
**A** Schematic of antitumor mechanism of CuCo_2_S_4_-Pt-PEG, (a) Illustration of the synthetic procedure of CuCo_2_S_4_-Pt-PEG, (b) Schematic of CuCo_2_S_4_-Pt-PEG with PTT/PDT/enzyme catalytic activity for synergetic therapy. Copyright 2024, Elsevier Ltd. [154]. **B** Schematic representation of the synthesis, trimodal imaging, and CDT-PTT of Ir@PLNPs@EM. Copyright 2024, Wiley–VCH GmBH [161]. **C** Schematic illustration for (a) the preparation of CPTH-AT nanozyme and (b) in vivo combined therapeutic of nanozymes-mediated biocatalytic immunotherapy and PTT. Copyright 2024, Elsevier Ltd. [162]. **D** (a) Synthesis of MLP@DHA&Ce6. (b) Schematic illustration of the mechanism behind MLP@DHA&Ce6-induced cell death, involving the generation of ROS from multiple sources [165]. Copyright (2023) American Chemical Society. **E** Schematic illustration of synergistic anti-tumor photodynamic therapy and immunotherapy based on CDK4/6 Nano PROTAC. Copyright 2024, Elsevier Ltd. [166]. **F** Synthesis and mechanism of action of Gd_2_O_3_/BSA@MoS_2_-HA, (a) Synthetic route of Gd_2_O_3_/BSA@MoS_2_-HA, (b) These nanoparticles could be applied for MSOT/CT/MR guided photothermal/radio combined cancer therapy, (c) After reaction with GSH, the NPs break down and are excreted out of the body. Reprinted with permission from [167]. Copyright (2024) American Chemical Society

Xu et al. constructed ultrasmall CuCo_2_S_4_-Pt-PEG nanocomposites with PTT/PDT enzyme catalytic activity for imaging-guided synergistic treatment, as shown in Fig. 10A [154]. CuCo_2_S_4_-Pt-PEG exhibits remarkable photothermal performance upon 1064 nm laser treatment with a low power density. The nanocomposites also possess multiple nanozyme activities (POD and CAT). CuCo_2_S_4_-Pt-PEG can disintegrate endogenous H_2_O_2_ to generate •OH, which induces tumor cell apoptosis due to its POD-like activity. Additionally, the nanocomposite can catalyze H_2_O_2_ into H_2_O and O_2_ through CAT-like catalytic activity, alleviating tumor hypoxia. Furthermore, compared to CuCo_2_S_4_-PEG, the CAT-like activity of Pt nanozyme enables CuCo_2_S_4_-Pt-PEG to produce more O_2_, resulting in a more pronounced PDT effect. The combination of CuCo_2_S_4_ and Pt mitigates the damage caused by overheating but also enhances the synergistic treatment capability.

PDT/PTT combinational strategy represents a noninvasive approach that synergistically enhances the efficacy of cancer therapies by integrating PDT and PTT [155, 156]. Effective phototherapy relies on three essential components: a photosensitizer, light of a specific wavelength, and adequate oxygen supply. However, the aggressive proliferation of tumors, coupled with limited blood supply, can result in insufficient oxygen levels within solid tumors, potentially limiting the effectiveness of phototherapy [157]. Therefore, nanozymes exhibiting enzyme-like activities, particularly those demonstrating peroxidase (POD) and catalase (CAT) characteristics, have been developed to address the issue of hypoxia in tumor parenchyma. This advancement contributes significantly to enhancing the efficacy of phototherapy.

Kothari et al. reported the effect of oxygen-generating nanozymes on indocyanine green and IR 820-mediated phototherapy against oral cancer [158]. CeO_2_ and Fe_3_O_4_ nanoparticles were produced using a surfactant-assisted one-pot hydrothermal approach, while MnO_2_ nanoparticles were created through a stoichiometric reduction method. The main goal of this work was to clarify how water-dispersible catalase-like metal oxide nanozymes influence the photodynamic effects of ICG and IR 820. The optimal combination of these nanoparticles, identified via Box-Behnken Design, was then assessed in the FaDu cell monolayer and spheroid models.

#### PTT/CDT and CDT/PTT

PTT/CDT cancer therapy refers to a combined treatment strategy that integrates photothermal therapy (PTT) and chemodynamic therapy (CDT) to enhance the effectiveness of cancer treatment. This dual-modal treatment capitalizes on the strengths of both approaches [159]. PTT elevates local temperatures, improving the generation of reactive oxygen species (ROS) during CDT. Additionally, the heat produced by PTT increases the permeability of tumor blood vessels, facilitating more effective delivery of CDT agents to the tumor site. This synergetic strategy targets cancer cells through various mechanisms, effectively addressing potential resistance while resulting in reduced side effects and enhanced treatment efficacy [160].

Li et al. and coworker developed a single-atom iridium nanozyme-@PLNPs for cancer theranostics [161]. PLNPs were prepared with the composite of Zn_1_.^2^Ga_1_.^6^Ge_0_. ^2^O_4_:Cr_0.0075_ for near-infrared (NIR) emission at 700 nm. The PLNPs were then incubated with an IrCl_3_ solution. After annealing at 750 °C, iridium was observed at the atomic level on PLNPs as a single-atom nanozyme with POD-like catalytic activity, photothermal capability, and computed tomography (CT) contrast capability due to the heavy atom property of iridium, as Ir@PLNPs. After coating with EM, the Ir@PLNPs@EM composite exhibited long-lasting NIR luminescence, peroxidase-like activity, photothermal conversion, and CT contrast capability, while EM improved biocompatibility and achieved targeting delivery (Fig. 10B). The Ir@PLNPs@EM composite effectively inhibited tumor growth through combined CDT and PTT under NIR afterglow/CT/photothermal trimodal imaging guidance. This is the first SAzyme@PLNP for theranostic applications and represents a significant advancement in developing specific, safe, and efficient multifunctional SAzyme@PLNPs for theranostics.

#### PTT/IT

The integration of PTT with immunotherapy combines the direct tumor-killing effects of PTT with the systemic immune activation provided by immunotherapy, enhancing therapeutic outcomes, increasing tumor immunogenicity, and potentially overcoming resistance mechanisms [147]. As research progresses, the PTT/immunotherapy combination holds significant promise for developing more effective and personalized strategies for cancer treatment. Overall, this combination represents a compelling strategy in cancer therapy, aiming to leverage the strengths of both modalities to improve patient outcomes and enhance the overall effectiveness of cancer treatments.

Lu et al. presented a multifunctional nanozyme (CPTH-AT) that exhibits structural similarity to the natural superoxide dismutase (SOD) [162]. This nanozyme can potentially target mitochondria, amplify mitochondrial oxidative stress through a cascade of enzyme activities mediated by biocatalysis, alleviate the immunosuppressive microenvironment, and enhance antitumor immune responses (Fig. 10C). In natural SOD enzymes, copper (Cu) serves as the catalytic site of the enzymatic reaction, undergoing a continuous cycle of reduction and oxidation in the presence of O_2_^•–^ to facilitate electron transport.

#### CDT/PDT

Synergistic Chemo-Dynamic Therapy (CDT) combined with Photodynamic Therapy (PDT) offers a groundbreaking method for treating cancer, using the benefits of both therapies to improve [163]. This collaborative approach uses nanomaterials in CDT to generate reactive oxygen species (ROS) when specific substrates are present, allowing for targeted damage to cancer cells [164]. Meanwhile, PDT uses photosensitizers activated by light to create ROS, which destroys tumors in a localized manner. Merging these therapies significantly boosts ROS production and enhances target specificity, reducing harm to nearby healthy tissues [160]. This comprehensive strategy has the potential to address resistance often seen in conventional treatments, enhance the effectiveness of tumor removal, and may lead to more favorable patient outcomes. By harnessing the strengths of CDT and PDT, this synergistic therapy represents a promising pathway for creating more effective and tailored cancer treatments.

Liu et al. introduced a lipid-based manganese oxide nanozyme, MLP@DHA&Ce6, for enhanced CDT/PDT via induction of multisource ROS [165]. This nanozyme is formed by a MnO_2_ nano-shell enclosed in a liposome, which carries dihydroartemisinin (DHA) along with the photosensitizer Ce6. It generates multiple sources of reactive oxygen species (ROS) to improve cancer treatment. MLP@DHA&Ce6 tends to accumulate in tumors while releasing key active substances such as Mn^2+^ ions and O_2_. The nanozyme facilitates the creation of ROS through several mechanisms: the nanozyme-catalyzed conjugated double-stranded DNA (CDT) reaction using DHA as a substrate, photodynamic therapy (PDT) involving Ce6, and the Fenton reaction driven by Mn^2+^ ions. The schematic illustration is shown in Fig. 10D.

#### PDT/IT

The combination of photodynamic therapy (PDT) and immunotherapy represents a promising strategy for cancer treatment. This integration has the potential to enhance the immune response to tumors. The cell death triggered by PDT can release antigens and activate immune cells, thereby strengthening the immune reaction. By synergistically using these two approaches, researchers aim to improve treatment effectiveness, reduce tumor burden, and establish a more comprehensive strategy for cancer management. Ultimately, this could lead to better treatment strategies and increased survival rates [166].

Wang et al. developed a CDK4/6 PROTAC and paired it with Chlorin e6 (Ce6)-mediated photodynamic therapy (PDT). PDT promotes highly targeted apoptotic cell death activated by localized light exposure and stimulates the intrinsic pathway of cell death (ICD), releasing various pro-inflammatory cytokines and modulating the infiltration of different immune cells into the tumor [20]. The PROTAC and Ce6 can spontaneously assemble into nanoparticles without additional carrier materials. This combination can potentially combat cancer and synergistically activate an anti-tumor immune response directly. This characteristic further supports co-delivery and enhances the concentration of both drugs within the tumor, thus improving their clinical applicability (Fig. 10E).

#### Photo/RT

Combined photo-radiotherapy represents an innovative cancer treatment strategy integrating phototherapy treatment approaches such as PDT or PTT with traditional radiotherapy. This approach aims to enhance the overall effectiveness of cancer treatment by harnessing the unique mechanisms of action inherent to both modalities, delivering a more comprehensive assault on tumors. Phototherapy's localized effects can make cancer cells more sensitive to radiation, potentially increasing the effectiveness of radiotherapy while reducing harm to nearby healthy tissues. Additionally, the immune response triggered by phototherapy could boost therapeutic outcomes, resulting in better tumor management and reduced chances of metastasis. As research in this area progresses, combined photo-radiotherapy shows significant promise for developing more effective and personalized cancer treatment regimens.

Cheng et al. developed ultrasmall Gd_2_O_3_ nanoparticles for Photothermal/radio combination therapy [167]. They conjugated clearable protein-based Gd_2_O_3_/BSA nanoparticles with MoS_2_ and modified with HA, resulting in distinct advantages. These nanoparticles can be used in combination therapy for cancer treatment, guided by multimodal imaging techniques such as MSOT/CT/MR. Figure 10F presents a detailed schematic illustration of this work. Furthermore, the liver can effectively eliminate protein-based nanoparticles. MoS_2_, linked via breakable disulfide bonds, was used in MSOT/CT imaging and photothermal/radiation therapy. Additionally, HA was introduced via breakable disulfide bonds to enhance the nanoparticles’ physiological stability and tumor-targeting efficacy.

#### SDT/CDT

SDT is a novel treatment modality that uses ultrasound stimulation to activate sonosensitizers to produce ROS, cavitation, bubbles, heat [1]. Given its deep tissue penetration, sonodynamic therapy (SDT) is generally more appropriate for clinical applications than PDT [168]. However, factors such as hypoxia and excess glutathione (GSH) can limit the effectiveness of SDT on its own [169]. Combining chemotherapeutic drug therapy with SDT can significantly enhance tumor treatment efficiency by promoting reactive oxygen species (ROS) production [170]. Recent research has also indicated that incorporating ultrasound can boost the activity of the Fenton reaction, thereby improving the overall effectiveness of CDT [171]. Given the considerable clinical potential of the SDT/CDT combination in managing deep-seated tumors, it is recognized as a promising bimodal therapy.

Feng et al. presented a novel Z-scheme heterojunction (HJ) sonosensitizer using Fe-doped carbon dots (CDs) as auxiliary semiconductors to sensitize cubic Cu_2_O (Fe-CDs@Cu_2_O) [172]. Fe-CDs@Cu_2_O exhibited enhanced sonodynamic therapy (SDT) effects due to improved electron–hole separation. Furthermore, introducing Fe ions in CDs synergistically enhances Fenton-like reactions with Cu ions in Cu_2_O, resulting in enhanced chemodynamic therapy (CDT) effects. Fe-CDs@Cu_2_O also exhibited rapid glutathione (GSH) depletion, effectively mitigating tumor microenvironment (TME) resistance.

Hu et al. reported the passivation and selective activation strategies for the sonodynamic and near-infrared (NIR) imaging performances of an intelligent antitumor theranostic platform, termed Cu-IR783 nanoparticles (NPs) [173]. The selective activation of IR783 in tumor tissues, facilitated by the ruptured coordination bond between IR783 and Cu ions in response to the tumor microenvironment (TME) enables the visualization of in-situ sonodynamic therapy (SDT). The tumor-specific release of Cu ions enhances the generation of ROS through Cu + -mediated Fenton-like reactions and induces cuproptosis by promoting DLAT oligomerization and causing mitochondrial dysfunction. Notably, the immunosuppressive TME can be reversed by the elevated ROS levels and high-efficiency cuproptosis, ultimately inducing immunogenic cell death that promotes robust systemic immune responses to eradicate primary tumors and suppress distant tumors.

### Triple therapy

#### PDT/PTT/CDT

In PDT and PTT, photosensitizers are activated by light exposure to induce cancer cell apoptosis [174–176]. In contrast, CDT operates through the generation of reactive oxygen species, leading to oxidative stress and subsequent damage to cancer cells. By combining the light-activated mechanisms of PDT and PTT with the ROS-generating capabilities of CDT, this therapy increases overall treatment efficacy while minimizing damage to surrounding healthy tissue. Nanozymes play a crucial role in enhancing the efficacy of trimodal cancer therapy by integrating PDT/PTT/CDT. Their unique properties allow for improved ROS generation, targeted delivery, and synergistic effects, making them a promising approach to fighting cancer. The trimodal strategy enhances the overall therapeutic effect and provides a more personalized and targeted approach to cancer treatment.

Hu et al. developed gold nanoshells decorated with platinum nanoclusters (Au NBPs@Pt NCs-ICG) that exhibit dual enzymatic activities to enhance multimodal imaging-guided phototherapy and chemodynamic therapy (CDT) [177]. However, these agents are distributed nonspecifically in the surrounding normal tissues, eventually damaging healthy cells. Specifically, guided photosensitizers with targeted recognition are crucial for effective biological diagnostics and therapies. For instance, Zhang et al. synthesized Au-Dsg-3 with dual-mode imaging for targeting lung SCC, facilitating precise diagnosis and targeted treatment to enhance synergistic PTT [178].

**Fig. 11:**
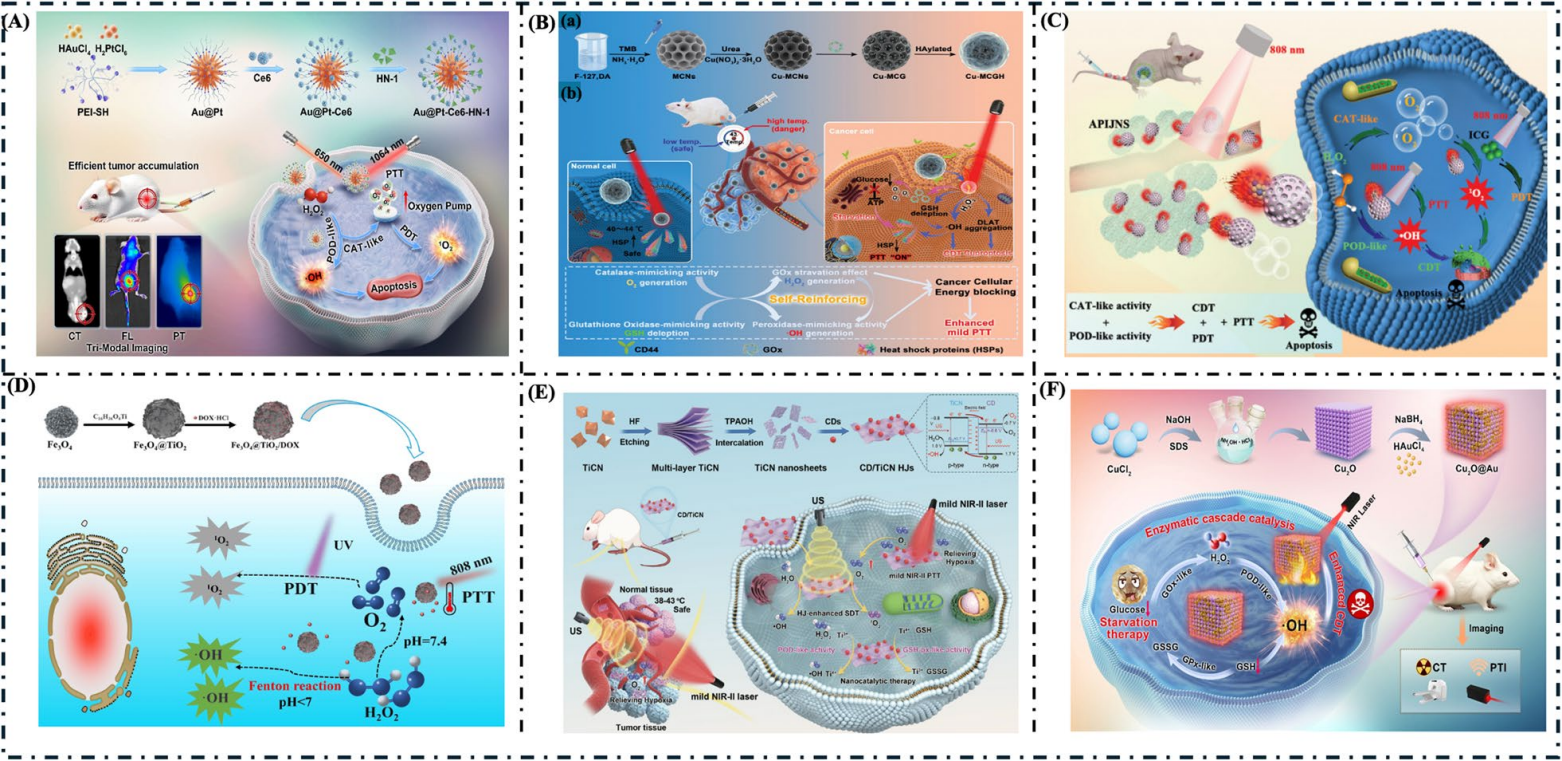
**A** Schematic illustrating the key steps in preparing the Au@Pt-Ce_6_-HN-1 nanoplatform and its multimodal imaging-guided synergistic PDT/PTT/CDT antitumor mechanism. Copyright 2024, Elsevier Ltd. [179]. **B** Schematic illustration of (a) the synthetic procedure and (b) the antitumor mechanism of the Cu-MCGH nanocomposite. Copyright 2024, Wiley–VCH GmBH [187]. **C** Schematic illustration of the parachute-like APIJNS synergistic CDT/PTT/PDT. Copyright 2024, Wiley–VCH GmBH [188]. **D** Schematic illustration of Fe_3_O_4_@TiO_2_/DOX microspheres and the combined CDT/PDT/PTT/chemotherapy for tumor inhibition. Copyright 2024, Nanomaterials [191]. **E** Schematic illustration of the sonoresponsive and NIR-II-photoresponsive CD/TiCN nanozymes for “three-in-one” multimodal oncotherapy. Copyright 2024, Wiley–VCH GmBH [194]. **F** Schematic diagram for the preparation of the Cu_2_O@Au nanozyme and the proposed antitumor mechanism [195]

Interestingly, Li et al. reported a dual-activity nanozyme as an oxygen pump to alleviate tumor hypoxia and enhance PDT/NIR-II PTT for sniping oral squamous cell carcinoma (OSCC) [179]. Figure 11A presents a schematic illustration of their reported approach, in which a meticulously crafted nanoplatform resembling a sea urchin composed of Au@Pt-Ce6-HN-1 is used to "snip" and eliminate OSCC. The Au@Pt-Ce6-HN-1 nanoplatform exhibits multifaceted functionalities, including catalase-like activity, peroxidase-like activity, and strong NIR-II. The platform also exhibits remarkable stability, biocompatibility, and exceptional tumor-targeting capabilities. It enables effective near-infrared-II-PTT and PDT treatments, offering a novel approach for developing nanoplatforms to precisely target tumors.

#### PTT/CDT/starvation therapy (ST)

The integration of PTT, CDT, and ST in a trimodal therapy framework exploits the synergistic effects of these modalities, enhancing overall treatment efficacy, reducing the likelihood of resistance, and improving cancer therapy outcomes. The responsive light sources of photosensitizers that currently enable PDT are primarily focused on ultraviolet and visible light, and their limited penetration depths significantly diminish the efficiency of PDT [180, 181]. Nanozymes are regarded as effective ROS generators [182]. Peroxidase (POD)-mimicking nanozymes can catalyze the overexpressed H_2_O_2_ to generate highly cytotoxic hydroxyl radicals (•OH) in the tumor microenvironment (TME) [183]. Chen et al. constructed an iron-based POD-mimicking nanozyme for in vivo tumor theranostics [184]. However, copper-based nanozymes can efficiently produce •OH over a broader pH range than the iron-catalyzed nanosystem [185]. Furthermore, the overloaded copper can induce the aggregation of lipoylated enzymes (such as dihydrolipoamide S-acetyltransferase, DLAT) and the loss of iron-sulfur cluster proteins (such as lipoyl synthase, LIAS) in the mitochondrial tricarboxylic acid cycle, leading to tumor cell cuproptosis [186]. Consequently, combining suitable NIR-II copper-based photothermal nanozymes and GOx may achieve maximized therapeutic efficacy through synergistic effects.

Bian et al. developed an enzyme-engineered coppery nanozyme for high-efficiency mild PTT/CDT/Starvation Therapy through self-reinforcing cancer energy metabolism regulation [187]. They report a straightforward strategy for the fabrication of an enzyme-engineered coppery nanozyme (named Cu-MCGH) based on dendritic mesoporous coppery carbon nanospheres as the foundation to deliver glucose oxidase (GOx) followed by modification with hyaluronic acid (HA), as depicted in Fig. 11B. Synthesized Cu-MCGH nanocomposite exhibits high GOx loading, strong NIR-II light absorption, and tumor-targeting capabilities. The concept of mild PTT/CDT/starvation combination therapy based on cuproptosis presents a promising strategy for potential tumor treatment, which has been applied to nanozyme-related therapeutic nanoplatforms in this study.

#### PTT/PDT/CDT

By combining PTT, PDT, and CDT, this integrated approach aims to exploit the synergistic effects of these therapies, leading to improved tumor destruction, reduced side effects, and enhanced overall treatment efficacy. The combination can also address various aspects of tumor biology, including metabolic vulnerabilities and the tumor microenvironment, ultimately improving patient outcomes in cancer therapy.

Zhang et al. reported Bimetallic nanozymes-integrated parachute-like Au_2_Pt@PMO@ICG Janus nanomotor with dual propulsion for enhanced tumor penetration and synergistic PTT/PDT/CDT cancer therapy [188]. They prepared a dual-source driven parachute-like Au_2_Pt@PMO@ICG Janus nanomotor (APIJNS) to enhance deep tumor penetration, alleviate tumor hypoxia, and augment the synergistic effect of PTT, CDT, and PDT. APIJNS exhibits high catalytic POD-like/CAT-like activity and a high photothermal impact. Notably, it efficiently catalyzes H_2_O_2_ to generate abundant reactive oxygen species (ROS) and achieves autonomous drive. It also forms a photothermally induced self-thermophoresis drive, thereby enhancing the PTT/PDT/CDT triple synergistic therapeutic effect. As depicted in Fig. 11C, under 808 nm near-infrared (NIR) laser irradiation, the exceptional and stable photothermal properties of Au_2_Pt nanozymes generate thermal gradients between the Au_2_Pt and PMO structures, facilitating self-thermophoresis propulsion. Concurrently, Au_2_Pt nanozymes possess a high CAT-like activity, enabling efficient H_2_O_2_ decomposition to produce O_2_, which subsequently serves as a fuel for photosensitizer ICG to enhance the efficacy of PDT. Furthermore, Au_2_Pt nanozymes exhibit highly efficient POD-like catalytic activity, catalyzing the decomposition of H_2_O_2_ to generate highly toxic •OH, thereby augmenting the hyperthermia-induced high-efficiency CDT in the tumor microenvironment (TME). Integrating dual-source autonomous driving mode, enhanced photothermal effect, and abundant ROS generation provides a novel perspective for the synergistic therapeutic effect of PTT/PDT/CDT. This research presents novel concepts for augmenting triple synergistic cancer therapy by enhancing tumor permeability by using dual-drive Janus nanomotors engineered to incorporate dual noble metal nanozymes.

#### CDT/PDT/PTT

The integration of CDT, PDT, and PTT within a trimodal therapy framework seeks to harness the synergistic effects of these treatment modalities [189]. CDT based on Fenton/Fenton-like reactions has recently garnered significant attention from researchers. This therapy exhibits remarkable properties, including high localization and selectivity, endogenous stimulation, low multidrug resistance, and enhanced effectiveness against tumor therapy. When combined with phototherapy, the efficacy of CDT is substantially augmented [190]. This synergistic approach enhances tumor ablation, reduces adverse effects, and improves treatment outcomes. By targeting various aspects of tumor biology and using multiple mechanisms of action, this approach can potentially address challenges commonly faced in traditional cancer therapies, such as drug resistance and tumor heterogeneity, ultimately leading to better patient treatment.

To improve the efficiency of PDT, Zhao et al. successfully prepared a novel composite photocatalytic material (Fe_3_O_4_@TiO_2_/DOX) composed of the typical photosensitizer TiO_2_ and enzyme Fe_3_O_4_ components (Fig. 11D) [191]. The Fe_3_O_4_ component’s catalase-like function can facilitate the breakdown of surplus H_2_O_2_, generating O_2_ within the tumor’s cytoplasm to improve PDT. Fe_3_O_4_ can slowly release Fe^2+^ and Fe^3+^, induced by the low-pH environment of tumor cells. H_2_O_2_ reacts with Fe^2+^ to form Fe^3+^ and hydroxyl radical to kill tumor cells. In detail, Fe^3+^ doping can transform GSH to glutathione disulfide (GSSH), decreasing ROS consumption Remarkably, the heat generated by the photothermal conversion of Fe_3_O_4_@TiO_2_ further promotes the efficiency of Fenton-like and photocatalysis reactions. The incorporation of DOX into Fe_3_O_4_@TiO_2_ microspheres enhances targeted delivery to cancer cells. Concurrently, these microspheres facilitate DOX's controlled and sustained release, optimizing therapeutic efficacy while mitigating potential side effects.

#### SDT/PTT/Nano catalytic therapy (NCT)

SDT/PTT/NCT utilizing nanozymes represents a cutting-edge approach in cancer treatment that synergistically combines SDT, PTT, and the catalytic properties of nanozymes to enhance therapeutic efficacy [132, 192]. Nanozymes, which mimic natural enzymes, can generate reactive oxygen species (ROS) under ultrasound or light stimulation, amplifying the oxidative stress on cancer cells. This trimodal therapy improves the precision of targeting tumor tissues and enhances the overall therapeutic effect by leveraging the unique advantages of each modality [116, 129]. Compared to traditional therapies, this approach offers improved selectivity, reduced side effects, and the ability to overcome drug resistance, making it a promising strategy for more effective and personalized cancer treatment.

Although nanozymes are widely used in nanocatalytic therapy (NCT), their limited catalytic activity and the complexities of the tumor microenvironment (TME) present significant challenges to the effectiveness of NCT. Additionally, creating a highly efficient and biodegradable nanozyme remains a major challenge, complicating their potential for clinical application. Interestingly, Hu et al. synthesize pH-responsive biodegradable CoSnO_3_ nanocubes, which are utilized as sonosensitizers and nanozymes due to their narrow bandgap of 1.6 eV and the multi-enzyme activities mediated by Co^2+^ and Sn^4+^ [193].

Recently, Zhao et al. and coworker, developed sonoresponsive and NIR-II-photoresponsive nanozymes based on the trimodal approach for heterojunction-enhanced three-in-one multimodal oncotherapy [194]. The TiCN nanosheets were synthesized through a modified liquid-phase exfoliation method involving two steps (Fig. 11E). NIR-II-photoresponsive nanozyme is assembled by attaching carbon dots (CDs) onto TiCN nanosheets. The narrow bandgap and mixed valences of Ti^3+^ and Ti^4+^ enable TiCN to generate reactive oxygen species (ROS) when exposed to ultrasound and the dual enzyme-like activities of peroxidase and glutathione peroxidase. The catalytic activities and sonodynamic properties of TiCN nanosheets are enhanced by the formation of heterojunctions, leading to faster carrier transfer and enhanced electron–hole separation. Introducing CDs with excellent NIR-II photothermal properties achieves mild hyperthermia (43 °C), further improving the NCT and SDT performances of CD/TiCN. The synergistic therapeutic efficacy of CD/TiCN through mild hyperthermia-amplified NCT and SDT can realize a “three-in-one” multimodal oncology therapy to eliminate tumors without recurrence.

#### ST/CDT/PTT

The integration of sonodynamic therapy (SDT), chemodynamic therapy (CDT), and photothermal therapy (PTT) using nanozymes represents a cutting-edge approach in cancer treatment, harnessing the unique properties of nanomaterials to enhance therapeutic efficacy. As research progresses, combining ST/CDT/PTT using nanozymes holds promise for the development of more effective and targeted cancer therapies, potentially improving patient outcomes and reducing treatment-related side effects.

Li et al. created a “tumor energy homeostasis disruptor," the Cu_2_O@Au nanozyme, which demonstrates exceptional glucose oxidase-like activity, allowing for its application in Starvation/CDT/PTT [195]. They developed a Cu_2_O@Au nanozyme as a “tumor energy homeostasis disruptor.” This innovative nanozyme integrates an ST approach with PTT-enhanced cascading catalysis of CDT as a synergistic therapeutic methodology (Fig. 11F). The Cu_2_O@Au nanozymes exhibit glucose oxidase (GOx)-like activity, consuming glucose at tumor sites to induce ST, thereby effectively severing the tumor cells’ energy supply. Concurrently, they continuously generate hydrogen peroxide (H_2_O_2_) and a more acidic tumor microenvironment (TME), amplifying CDT efficacy. Furthermore, the Cu_2_O@Au nanozymes display remarkable peroxidase (POD)-like activity, facilitating a Cu + -mediated Fenton-like reaction that transforms H_2_O_2_ at tumor locations into toxic hydroxyl radicals (•OH), consequently inducing cancer cell apoptosis. Simultaneously, Cu_2_O@Au nanozymes exhibit glutathione peroxidase (GPx)-like activity, persistently depleting glutathione (GSH) levels. These nanozymes also activate the p53 signaling pathway, resulting in enhanced expression of p53 and a concomitant decrease in SLC7A11 (xCT) protein levels. This reduction in xCT protein subsequently leads to diminished cystine uptake by the cancer cells. Furthermore, the Cu_2_O@Au nanozymes disrupt the tumor’s antioxidant defense system by modulating glutamate dehydrogenase 2 (GLS2) levels and nicotinamide adenine dinucleotide phosphate (NADPH). This further inhibits the activity of GPX4, obstructing reactive oxygen species (ROS) clearance and ultimately fostering the accumulation of lipid peroxides (LPO), thereby enhancing ferroptosis. The Cu2O@Au nanoparticles also demonstrate excellent photothermal properties. Under 808 nm laser irradiation, they facilitate an accelerated production of •OH while maintaining good photothermal performance and X-ray attenuation capacity, thereby rendering them suitable for photothermal imaging (PTI) and computed tomography (CT) imaging applications. Consequently, this study underscores the effectiveness of the Cu2O@Au nanozyme as a disruptor of tumor energy homeostasis, initiating cascade reactions with substantial therapeutic outcomes, thereby achieving multilevel tumor suppression intervention. Table 2 provides a detailed summary of various cancer therapies using nanozymes.

**Table 2:** Summary of nanozymes used for cancer therapy

Therapeutic modality	Types of therapy	Nanozymes	Functions	References
Monomodal therapy	Chemotherapy	CeO_2_-CuO NZ^a^	CAT^a^-like activity	[196]
		AuRu NZ^a^	CAT-like activity	[197]
		Co-SA/NC	CAT-like activity	[198]
	Photothermal Therapy	IB/Fe-ZIF8/PDFA	POD^a^-like activity	[199]
		Fe_3_O_4_/Cu_1.77_Se		[200]
		MoCu-Dazyme	OXD^a^-like activity	[201]
		ABZ/DOX/HA	POD, Gox-like activity	[202]
		MnZ-Au	CAT-like activity	[203]
	Photodynamic Therapy	MIL-53/cMBP/ST/Ce6	OXD-like activity	[117]
		ZMRP/HA	CAT, GSHOx^a^ mimic activity	[204]
		PMOF-HA	CAT-like activity	[205]
		GCS-I-PPy NZs	CAT-like activity	[206]
	Chemodynamic Therapy	ZIF-8/SrSe/DOX	GSHOx mimic activity	[207]
		MNZs	GSH-OXD-like function	[123]
		Au/CeO_2_ NZ^a^	Glucose-oxidase mimic	[208]
		Au–Pt NZ^a^	CAT mimic	[209]
		ZnO_2/_Pt	POD-like activity	[124]
	Sonodynamic Therapy	Fe–Ni–CDs	POD-like activity	[210]
		PdCux/LDH	OXD and CAT-like activities	[133]
		GQD/CoSnO_3_	POD-mimic catalytic reaction	[193]
		Pt/PCN-224(Fe)/PEG	Glutathione peroxidase (GPX)	[134]
		HABT-C NPs	POD and COD-like activity	[127]
		DPC/Pt/M	SOD-CAT, SOD-POD	[211]
	Immunotherapy	PBAF	POD and GSHOx-like activity	[212]
		Cu-NS/UK/Pox	OXD, POD, GSHOx-like activity	[213]
		SS-MSN/Au-BOM	PODand CAT-like activity	[143]
		N-PCNS	POD-like activity	[214]
	Starvation Therapy	ZIF/HAgel-GOx	OXD-like activity	[145]
		Cu–Ag NP^a^	OXD-like activity	[146]
		HMnO_2_	OXD-like activity	[144]
Bimodal therapy	PTT/PDT or PDT/PTT	Au@Pt-Ce6-HN-1	CAT-like activity	[179]
		CeO_2_, Fe_3_O_4_, and MnO_2_	CAT-like activity	[158]
		CHC	POD-like activity	[215]
		CCC	CAT, POD, OXD, and GSHOx-like activities	[216]
		Wheel-shaped POMs		[217]
		IR780-HCuS/MnO_2_	CAT-like activity	[218]
		PtBi-β-CD-Ce6	CAT-like activity	[219]
	PTT/CDT or CDT/PTT	MLP/DHA&Ce6	CAT-like activity	[165]
		SnFe_2_O_4_, SFO NZ^a^	CAT-like activity	[220]
		PVP/MnO_2_-Ti3C_2_		[221]
		AuMnCu, AMC	CAT, POD, and GSHOx-like activities	[222]
	PTT/IT or IT/PTT	Cu-based NZ^a^	CAT-like activity	[223]
		Fe-MOF-RP	POD-like activity	[118]
		CMO-R/4T1	POD-like activity	[224]
	CDT/PDT or PDT/CDT	MNPs/GOD-CS/IR820	CAT and POD-like activity	[225]
		Ce6/CMNR	CAT-like activity	[226]
		Au-Co_3_O_4_ NZ	SOD^a^, CAT-like activity	[227]
	PDT/IT or IT/PDT	ZnO-Ce6 NPs	CAT-like activity	[228]
		ZnPc/FOM-Pt		[229]
	CDT/IT or IT/CDT	HE-LDH	SOD, POD and GPX-like activity	[230]
		COF-derived carbon NZ^a^	POD and OXD-like activity	[231]
	SDT/IT or IT/SDT	Fe-PDAP/Ce6	CAT-like activity	[232]
		SAEs		[233]
	CDT/SDT or SDT/CDT	ZnO_2_/Au/ZIF-67 NPs	CAT-like activity	[234]
		Fe-CDs/Cu_2_O	GSH depletion	[172]
		ICG/Met-H-MnO_2_/MPN-FA (IMMMF)	CAT-like activity	[235]
	ST/CDT or CDT/ST	H-CeO_2_/Ce6/PDA/GOx NPs	CAT-like activity	[159]
		CaCO_3_/MnO_2_-NH_2_/GOx/PVP (CMGP)	CAT-like activity	[236]
	SDT/ST or ST/SDT	AuPt/MgSiO_3_/GOx, APMS-Gox	CAT-like activity	[237]
	PTT/ST or ST/PTT	AuPtAg NZ^a^	CAT-like activity	[238]
		FePGOGA NZ^a^	POD and GSHOx-like activity	[239]
	PDT/ST or ST/PDT	Ce6/HGMOF	CAT-like activity	[240]
Trimodal therapy	PDT/PTT/CDT	Fe_3_O4/TiO_2_ microspheres	POD, CAT-like activity	[191]
		CeO_2_/ICG/GOx/HA	OXD, CAT, SOD, POD-like activity	[241]
	PTT/CDT/ST	Fe_2_O_3_/Au hybrid NZ^a^	POD-like activity	[153]
		Cu-MCGH NC^a^	POD and GSHOx-like activity	[187]
	PTT/PDT/CDT	Au NBPs/Pt NCs-ICG	CAT and POD-like activity	[177]
		PBCI	POD and CAT-like activity	[242]
		CoPc-Mn/Ti^3^C_2_Tx	SOD-mimicking activity	[243]
		Fe_3_O_4_/Au NCs/L-CPAA-TPP	CAT-like activity	[244]
		APIJNS	CAT-like activity	[188]
	CDT/PDT/PTT	HA-ICG-Fe-PDA	Fenton-like reaction	[245]
		FeNC/PAA NP^a^	Fenton-like reaction	[246]
		MOF/CuS NPs	Fenton-like reaction	[163]
	SDT/PTT/NCT	CuS NPs	POD, SOD, CAT-like activity	[247]
		CD/TiCN	POD, GSHOx-like activity	[194]
	CDT/Chemo/ST	TPZ/Fe_3_O_4_/MSN-GOX	Fenton-like reaction	[248]
	CDT/PDT/IT	USPFLR nano-bomb	Fenton-like reaction	[249]
	SDT/CDT/ST	PtMo-Au metalloenzyme	CAT-like activity	[250]
	PDT/CDT/PTT	MnO_2_/Ag_3_SbS_3_ NPs	Fenton-like reaction	[251]
	PTT/PDT/ST	BP/Au/MnO_2-_PEG	Glucose-like oxidase activity	[252]

^a^Where *NZ* nanozyme, *NP* nanoparticles, *NC* nanocomposite, *CAT* catalase, *POD* peroxidase, *SOD* superoxide dismutase, *OXD* oxidase, *GSHOx* glutathione-oxidase-like

## Current challenges and prospects

Nanozymes have demonstrated potential for tumor therapy, but further research is needed to overcome the limitations associated with their current state of development. Despite significant advancements in cancer treatment through nanozymes, there remains substantial room for further growth in this field. In this regard, we make the following suggestions to address some of the challenges encountered during the current research process of nanozymes, seeking to enhance the broader application of nanozymes in cancer treatment.

First, nanoenzymes can be used as imaging agents to detect cancer and monitor treatment responses. Investigating their potential in diagnostic applications, such as tumor imaging and biomarker detection, can significantly expand their role in oncology.

Second, during the development of nanocatalytic medicine, ROS are one of the key factors in nanomedicine therapy. Nanomaterials are utilized to catalyze intracellular oxygen or oxygen molecules to generate excessive ROS to induce cells apoptosis. At present, the applications of nanozymes in biomedicine mainly focus on the simulation of catalase, peroxidase, and oxidase. A variety of nanocatalytic drugs that have unique responses to endogenous or exogenous physical stimuli, such as sensitizers, photosensitizers, and photothermal agents, are applied to activate or assist the generation of ROS for a high level of activity.

Third, different enzymes could catalyze the same substrate, and there is a competitive relationship. For instance, since catalase and superoxide dismutase can catalyze the decomposition of hydrogen peroxide, a competitive relationship exists during the process of catalyzing hydrogen peroxide. Consequently, catalase or superoxide dismutase in the body competes with nanozymes specifically designed to mimic catalase and superoxide dismutase. This competition may result in the simultaneous or sequential impact of natural enzymes and nanozymes, potentially affecting their efficacy. Consequently, designing nanozyme structures to mitigate competitive effects and subsequently harness their potential remains an area of ongoing research.

Fourth, enhancing the stability, biocompatibility, and selectivity of nanozymes within living organisms and their capability for tumor targeting is paramount. Surface modification is a typical approach for preparing nanozymes. Nevertheless, it might obstruct the interaction between the nanozyme surface and the substrate, leading to less than the optimal catalytic performance. Therefore, it is essential to optimize the surface modification techniques and devise a strategy that reduces the impact of surface modification on nanozyme activity.

Fifth, investigations into biochemistry and clinical outcomes reveal that tumor heterogeneity significantly impacts the effectiveness of certain nanozymes during experimental trials. This variability in tumor characteristics means that not all nanozymes can achieve the desired effects. As a result, the design and development of effective nanozymes pose ongoing challenges for researchers in the field. Preclinical studies have shown the potential of nanozymes in cancer treatment; however, translating these discoveries into clinical practice faces significant challenges. It is essential to address regulatory and safety concerns, as well as to streamline manufacturing processes, to accelerate this translation. Achieving this goal requires a thorough and comprehensive research effort.

To overcome these challenges, ongoing research is focused on optimizing the design and functionalization of nanozymes to increase their biocompatibility, improve their catalytic efficiency, and increase their specificity for cancer cells. Addressing these issues is crucial for successfully translating nanozyme technology into effective cancer therapies. Overall, the future of nanozymes in therapeutics holds great promise, with the potential to significantly improve patient outcomes and revolutionize the landscape of modern medicine.

## Conclusion

In conclusion, we provide a comprehensive overview of nanozymes, highlighting their unique properties, various classifications, catalytic activities, and diverse applications in cancer treatments. Nanozymes hold great promise for therapeutic applications, marking an exciting advancement in the development of treatments for cancer. Their unique enzyme-like properties enable them to generate reactive oxygen species (ROS) that can selectively induce oxidative stress in cancer cells, leading to cell death while minimizing damage to surrounding healthy tissues. The stability, cost-effectiveness, and tunable catalytic activities of nanozymes make them particularly advantageous compared to traditional therapeutic agents.

We have summarized the recent advancements in nanozymes as smart treatment nanoplatforms for effective tumor therapy through single therapy such as, chemotherapy, phototherapy (PTT and PDT), chemodynamic (CDT), sonodynamic therapy (SDT), starvation therapy (ST), immunotherapy (IT), and dual therapy methods. Furthermore, integrating nanozymes into multimodal treatment strategies, such as combining them with PDT, PTT, CDT, and SDT, enhances their therapeutic efficacy and provides a synergistic effect that can overcome some of the limitations associated with conventional cancer treatments. As research advances in the design and synthesis of novel nanozymes, their potential applications in cancer therapy are expected to expand further. Future studies should focus on optimizing their delivery systems, enhancing their selectivity, and exploring their roles in personalized medicine. Overall, nanozymes hold great promise as a versatile and effective tool in the ongoing fight against cancer, paving the way for innovative therapeutic strategies that could significantly improve patient outcomes.

## Data Availability

No datasets were generated or analysed during the current study.
